# Trans-differentiation of Jdp2-depleted Gaba-receptor-positive cerebellar granule cells to Purkinje cells

**DOI:** 10.1038/s41420-024-02262-2

**Published:** 2024-12-18

**Authors:** Chia-Chen Ku, Jia-Bin Pan, Kenly Wuputra, Wen-Li Hsu, Kohsuke Kato, Michiya Noguchi, Yukio Nakamura, Shigeo Saito, Cheng-Yu Tsai, Ying-Chu Lin, Deng-Chyang Wu, Chang-Shen Lin, Kazunari K. Yokoyama

**Affiliations:** 1 Graduate Institute of Medicine, Kaohsiung Medical University, Kaohsiung, Taiwan; 2 Regenerative Medicine and Cell Therapy Research Center, Kaohsiung Medical University, Kaohsiung, Taiwan; 3 Cell Therapy and Research Center, Kaohsiung Medical University Hospital, Kaohsiung, Taiwan; 4 Department of Dermatology, Kaohsiung Municipal Ta-Tung Hospital, Kaohsiung Medical University Hospital, Kaohsiung, Taiwan; 5 Department of Infection Biology, Graduate School of Comprehensive Human Sciences, the University of Tsukuba, Tsukuba, Japan; 6 Cell Engineering Division, RIKEN BioResource Research Center, Tsukuba, Ibaraki Japan; 7 Saito Laboratory of Cell Technology, Yaita, Tochigi Japan; 8 Division of Neurosurgery, Department of Surgery, Kaohsiung Medical University Hospital, Kaohsiung, Taiwan; 9 Division of Neurosurgery, Department of Surgery, Kaohsiung Medical University Gangshan Hospital, Kaohsiung, Taiwan; 10 Department of Post-Baccalaureate Medicine, Kaohsiung Medical University, Kaohsiung, Taiwan; 11 School of Dentistry, Kaohsiung Medical University, Kaohsiung, Taiwan; 12 Division of Gastroenterology, Department of Internal Medicine, Kaohsiung Medical University Hospital, Kaohsiung, Taiwan; 13 Department of Biological Sciences, National Sun Yat-sen University, Kaohsiung, Taiwan; 14 Present Address: National Center for Geriatrics and Welfare Research, National Health Research Institutes, Yunlin County, Taiwan

**Keywords:** Gene expression, Cellular neuroscience

## Abstract

The Jun dimerization protein (*Jdp2*) gene is active in mouse cerebellar granule cells and its protein product plays a crucial role in the formation of the cerebellum lobes through programmed cell death. However, the role of Jdp2 in cellular differentiation and pluripotency in the cerebellum, and the effect of the antioxidation reaction on cell plasticity, remain unknown. *N*-acetyl-l-cysteine (NAC) induced the early commitment of the differentiation of granule cell precursors (GCPs) to neurons, especially Purkinje cells, via the γ-aminobutyric acid type A receptor α6 subunit (Gabra6) axis; moreover, Jdp2 depletion enhanced this differentiation program of GCPs. The antioxidative effect of NAC was the main driving force of this decision toward the neural differentiation of the GCP population in the presence of Gabra6 in vitro. This implies that antioxidative drugs are effective agents for rescuing oxidative-stress-induced GCP damages in the cerebellum and commit this Gabra6-positive cell population toward differentiation into Purkinje cells.

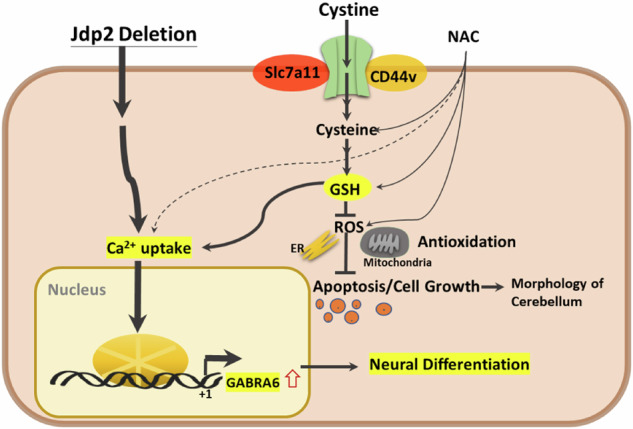

## Introduction

Many neurodegenerative diseases included Alzheimer’s disease, Huntington’s disease (HD), Parkinson’s disease (PD), spinocerebellar atrophy, spinal muscular atrophy, and amyotrophic lateral sclerosis are characterized by the progressive loss of function and death of specific neurons that give rise to the clinical manifestation of the disease [1]. In general, the stem cells were sequentially differentiated into various kinds of neurons and glia with an effort made to mimic the process of development of the nervous system. Thus, the methods of the neural cell progenitors to differentiate or transdifferentiate into neuros should be developed and examined their preclinical application for these neurodegenerative diseases.

In some case, the alterations in specific proteins in neural generative diseases lead to dysfunction of different cellular pathways, including increased numbers of reactive oxygen species (ROS) derived from mitochondrial dysfunction, excitotoxicity, synaptic dysfunction, impairment of protein degradation, endoplasmic reticulum stress, DNA damage, inflammation, and cell cycle reentry [2]. The neural tissue is highly sensitive to oxidative stress, and this is a prominent factor in both chronic and acute neurodegeneration. Based on this knowledge, therapeutic strategies using antioxidant molecules towards redox equilibrium have been widely used for the treatment of several brain pathologies [3, 4].

Granule cell progenitors (GCPs) are the most abundant type of cells in the mammalian brain [5–7]. The generation of GCPs from embryonic stem cells (ESCs) has contributed remarkably to the control of the in vitro differentiation of a variety of neurons [8, 9]. The techniques used to differentiate GCPs into specific neuronal cells, such as Purkinje cells (PCs), enable the establishment of in vitro cell-based models as platforms for drug discovery and preclinical translational research.

Previously, we demonstrated that the animals derived from Jun dimerization protein 2 (*Jdp2*)*-Cre*/ZEG lines expressed green fluorescence protein signals in the brain, predominantly in the cerebellum. Moreover, β-Gal staining revealed that the signals were localized in the cerebellum of *Jdp2-Cre*/ZEG mice. In the absence of Jdp2, a complex of the cyclin-dependent kinase inhibitor 1 (p21^Cip1^) and Nrf2 bound to the antioxidant response elements of the *Slc7a11* promoter and provided redox control to block ROS-mediated apoptosis [10, 11]. However, the role of Jdp2 in the neural differentiation of GCPs remains unknown.

It has been reported that mouse ESC-derived serum-free floating culture of embryoid-body-like aggregates can lead to their differentiation into PCs [12]. In a three-dimensional human ESC culture, polarized cerebellar self-organized neuroepithelial cells differentiated into PCs [13–15]. Granule cells (GCs) or GCPs can influence PC development from the moment PCs migrate in the primordial cerebellum to form the cerebellar networks [16]. γ-aminobutyric acid (Gaba) depolarized GCPs *via* Gaba type A (GabaA) receptors and led to calcium increases in GCPs [17]. In turn, loss of PCs in the cerebellum is a characteristic of dominantly inherited neurodegenerative diseases, such as spinocerebellar ataxia type 6 (SCA6). We investigated this protocol for the differentiation of GCPs to PCs and generated primary cultures of the sorted subpopulations of Gaba type A receptor α6 subunit (Gabra6)-positive (Gabra6^+^) GCPs from wild-type (WT) and *Jdp2* knockout (KO) mice, then compared their proliferation and differentiation activities in the presence or absence of *N*-acetylcysteine (NAC), an antioxidation reagent, after triggering the cell-differentiation program of GCPs into the neurons. Here, we observed that most of the sorted Gabra6^+^ GCPs transdifferentiated into calbindin-positive PCs in the presence of NAC in our in vitro culture condition. Thus, we concluded that Jdp2 is a critical regulator that contributes to the blockage of the differentiation of Gabra6^+^-sorted GCPs into various types of neurons, especially PCs, which are of vital importance in the context of neurodegenerative diseases [18]. Taken together, our findings suggest that NAC induces the neural differentiation of GCPs, and that the deletion of *Jdp2* enhances this differentiation program in Gabra6-sorted GCPs. This finding may contribute to the development of new therapeutics for the differentiation of predominant GCPs to PCs, to cure neurodegenerative disorders.

## Results

### Isolation of the purified Gabra6-positive subpopulation of GCPs

**Fig. 1:**
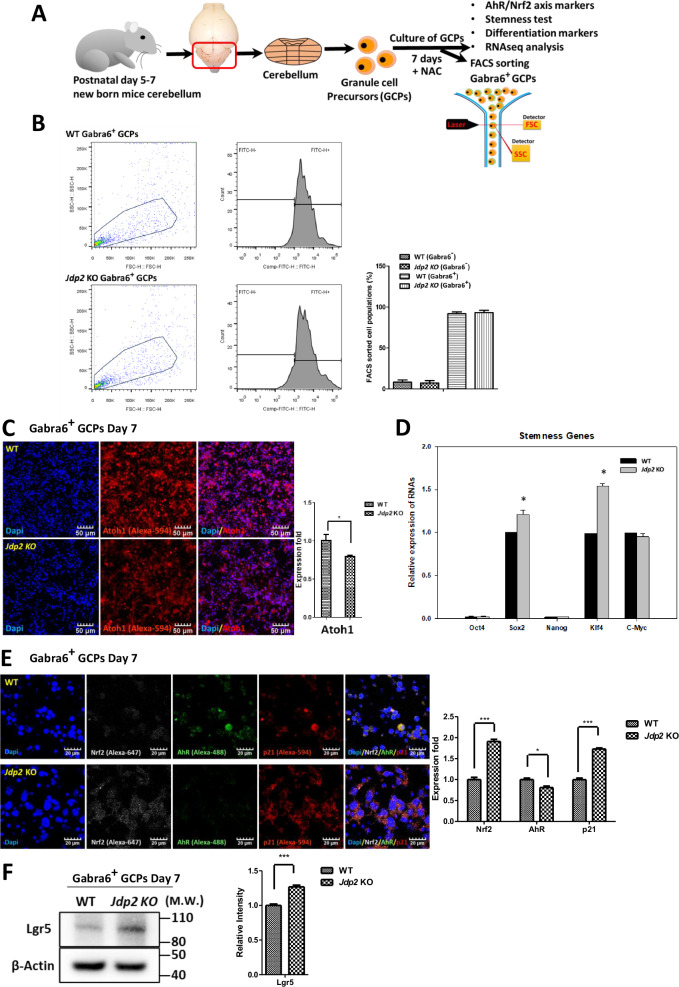
Characterization of mouse Gabra6^+^ GCPs by fluorescence-activated cell sorting (FACS). **A** Schematic model of the cultivation of mouse GCPs purified via discontinuous density gradient centrifugation from the cerebella (postnatal days 5–7) of mice [15, 16] in the presence of NAC (30 μM) for 7 days [10, 11], followed by characterization and FACS-based sorting using an anti-Gabra6 antibody. **B** FACS-based sorting to enrich the subfraction of Gabra6^+^ GCPs from WT and J*dp2*KO mice. The left panel depicts a representative dot plot of FSC and SSC. The region was gated in dot plots and Gabra6^+^ cells were sorted by FACS analysis. The histogram was gated on a peak, to identify the percentage of GCPs that expressed Gabra6. **C** Staining of Gabra6^+^ GCPs from WT and *Jdp2* KO mice with an anti-Atoh1 antibody. The nuclei of PGCs were stained with DAPI. Relative ratio of Atoh1-positive cells among Gabra6^+^ GCPs from WT and *Jdp2* KO cells. Scale bars, 50 μm. **D** Relative values of the Oct4, Sox2, Nonog, Klf4, and c-Myc mRNAs between WT and *Jdp2* KO Gabra6^+^ GCPs. The primer sequences used for qPCR are listed in Supplementary Table 2. The levels of the WT cells were taken as 1.0. **E** Immunostaining of Gabra6^+^ GCPs from WT and *Jdp2* KO mice for Nrf2, p21^Cip1^, and AhR. The GC nuclei were stained with DAPI. The levels of the WT cells were taken as 1.0. Scale bars, 20 μm. **F** Comparative expression of the Lgr5 proteins, as assessed by Western blotting (left panel; the quantitation of each protein is shown on the right panel). The relative value was normalized to that of β-actin and presented as a ratio. Uncropped raw data was shown in Fig. S5. **B**–**F**: *n* = 5; *P < 0.05, **P < 0.01; ***P < 0.001.

RNA sequencing of cell-type marker genes of the cerebellum after the cultivation of GCPs in the presence of NAC for 7 days was performed [10] (Fig. S1A). These values (*P* < 0.05) were summarized relative to the total number of cells. When the expression levels of these markers in each representative cell population of *Jdp2-KO* GCPs were compared with those detected in WT GCPs, the expression of granule cell marker genes as the dominant population was lower than that observed in WT GCPs; in contrast, the expression of the marker genes of PCs, astrocytes, and oligodendrocytes was 1.7–2.0-fold higher in *Jdp2-KO* GCPs compared with WT GCPs after cultivation with NAC. These data are similar to the results obtained from the recent single-cell RNA sequencing reported by Carter et al. [19]. In addition, the gamma-aminobutyric acid A receptor (*Gabra*) subunit alpha 6 (*Gabra6*) mRNA has been reported to be expressed in the cerebellum during development [20]. Thus, we examined the expression levels of proteins such as PC-related and calcium-channel-related molecules in the presence of NAC for 7 days using Western blotting (Fig. S1B). The expression of Cacn alpha1a (1.4-fold), calbindin (1.3-fold), Gabra1 (1.3-fold), Gabra6 (1.5-fold), Gabrb2 (1.3-fold), Grin2a (1.2-fold), Pcp4 (2.0-fold), and Vglut1 (1.8-fold) was significantly upregulated in *Jdp2* KO vs. WT GCPs, indicating that Jdp2 might be the master regulator of Gaba-receptor-mediated neural differentiation into PCs [11]. Thus, we sorted the GCPs to isolate the Gabra6^+^ subpopulation after cultivation in the presence of NAC for 7 days using an anti-Gabra6 antibody (Fig. 1A, B); we found that more than 90% of the total cells were positive for Gabra6, whereas less than 5%–10% were negative for Gabra6 (Fig. 1B). These Gabra6^+^ GCPs were analyzed further for stemness, pluripotency, cell cycle, differentiation, and oxidation/antioxidation.

### Expression of genes related to stemness, differentiation, and the AhR–Nrf2 axis

For their characterization, the FACS-separated Gabra6-positive GCPs (Gabra6^+^ GCPs) were obtained after the cultivation of GCPs for 7 days in the presence of NAC and were then stained with an anti-Atoh1 antibody [10]. We found that Atoh1 expression was higher in WT compared with *Jdp2* KO cells (Fig. 1C). The previous results of an RNA sequencing experiment in cerebellar cells derived from P6 mice showed that more than 95% of cells were positive for Atoh1 [10]. After cultivation with NAC for 7 days, the number of Atoh1-positive cells decreased to 70%. In fact, 70% of Gabra6^+^ GCPs remained positive for Atoh1 (data not shown). Furthermore, stemness markers such as Sox2 and Klf4 were upregulated in *Jdp2* KO compared with WT cells. In turn, the levels of markers such as Oct4, Nanog, and c-Myc were lower, but not altered between WT and *Jdp2* KO cells with NAC for 7 days (Fig. 1D). Western blotting also confirmed this finding (data not shown). Moreover, the WT and *Jdp2* KO Gabra6^+^ GCPs were negative for alkaline phosphatase after cultivation in the presence of NAC for 7 days (data not shown). This indicates that cultivation with NAC resulted in the loss of the stemness character. The antioxidation-specific transcription factor Nrf2 and the cell-cycle regulator p21^Cip1^ were expressed at 1.9-fold and 1.7-fold higher levels in *Jdp2* KO vs. WT cells, respectively. In contrast, the oxidative-stress-related factor AhR was expressed at 81% lower levels in *Jdp2* KO compared with WT cells (Fig. 1E). The expression of Lgr5 in *Jdp2* KO was higher by 1.2-fold than that detected in WT cells (Fig. 1F). Thus, the addition of NAC to Gabra6^+^ GCPs seems to initiate the differentiation program while maintaining the stemness character.

### Cell proliferation and antioxidation

**Fig. 2:**
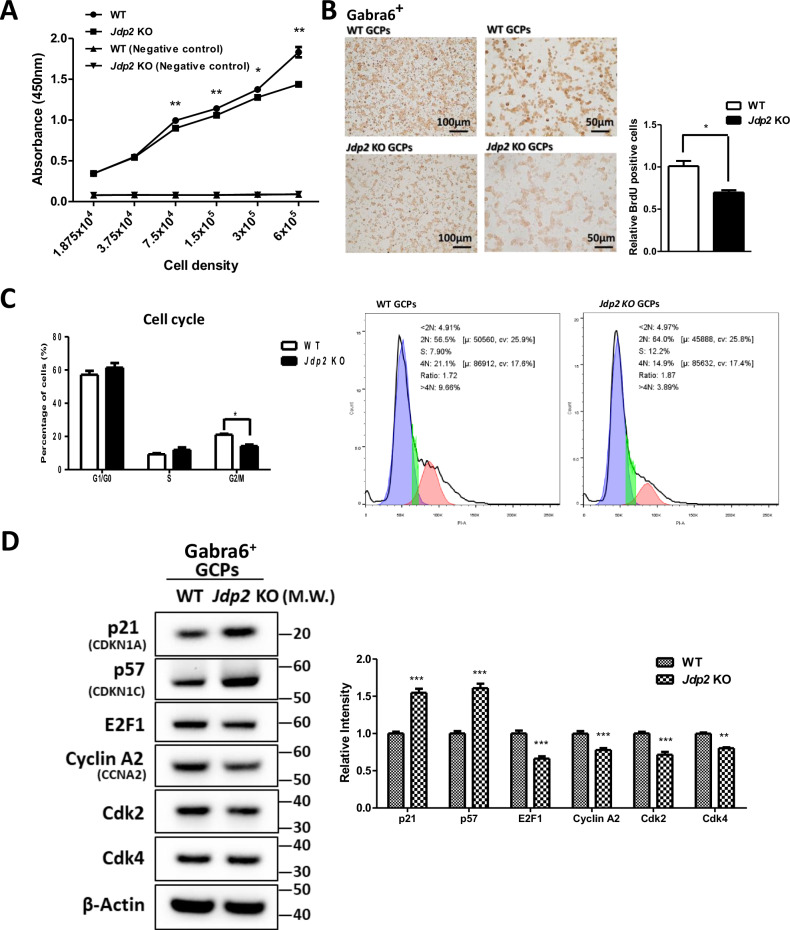
Comparison of the proliferation abilities and cell cycle regulation of Gabra6^+^ GCPs from WT and *Jdp2* KO mice. **A** Comparative incorporation assay of BrdU into the GCPs obtained from WT and *Jdp2* KO mice. **B** Immunocytochemical analysis of Gabra6^+^ GCPs from WT and *Jdp2* KO mice using anti-BrdU antibodies. Scale bars, 100 and 50 μm. Quantitation of the number of BrdU-stained GCPs from WT and *Jdp2* KO mice. **C** The cell-cycle analysis of WT and *Jdp2* KO Gabra6^+^ GCPs. The percentage of cells in the G_2_/M phase in *Jdp2* KO Gabra6^+^ GCPs was lower by 70% than that observed in WT Gabra6^+^ GCPs. **D** Western blot analysis of the comparative expression of cell-cycle-related proteins in WT and *Jdp2* KO Gabra6^+^ GCPs. The relative value was normalized to β-actin and presented as a ratio. The quantitation of relative expressions is summarized in the panels on the right. The levels of expression of all markers were significantly higher in *Jdp2* KO vs. WT Gabra6^+^ GCPs. (All data were obtained, *n* = 5: *P < 0.05, **P < 0.01; ***P < 0.001). Uncropped raw data was shown in Fig. S5.

The BrdU incorporation activity of WT GCPs was higher than that of *Jdp2* KO Gabra6^+^ GCPs (Fig. 2A). Moreover, immunocytochemistry demonstrated that the number of BrdU-positive Gabra6^+^ GCPs from WT mice was 1.4-fold higher than that from *Jdp2* KO mice (Fig. 2B), suggesting that Jdp2 is an activator of the proliferation of Gabra6^+^ GCPs. In addition, a comparative cell-cycle analysis between WT and *Jdp2* KO Gabra6^+^ GCPs showed that *Jdp2* KO triggered a concomitant decrease in the number of cells in the G_2_/M phase (by 70%) (Fig. 2C). Furthermore, Western blotting revealed that cell-cycle-arrest–related proteins, such as p21^Cip1^ and p57^Kip2^, were expressed at 1.5-fold and 1.6-fold higher levels, respectively, in *Jdp2* KO compared with WT cells. In contrast, cell-cycle-processing factors, such as E2F1, cyclin A2, Cdk4, and Cdk2, were decreased by about 60%–80% in *Jdp2* KO vs. WT cells (Fig. 2D). These data suggest that Jdp2 plays a critical role in the control of the cell cycle in Gabra6^+^ GCPs. In addition, our previous studies demonstrated that the apoptotic activity of WT GCPs was higher than that of *Jdp2* KO GCPs [11].

To clarify the mechanism underlying the upregulation of GSH in Gabra6^+^ GCPs in the presence of NAC, we examined the effect of NAC in WT and *Jdp2* KO Gabra6^+^ GCPs, to compare their antioxidation activities. Under the NAC condition, the levels of ARE-luciferase activity in WT and *Jdp2* KO cells were increased by 1.4–3.3-fold vs. those recorded in the absence of NAC (Fig. S2A). These results suggest that NAC plays a critical role in the antioxidation response; this effect was more sensitive in *Jdp2* KO Gabra6^+^ GCPs compared with WT Gabra6^+^ GCPs.

The expression levels of Gsk3β [21] and NeuN [22] as neural markers were significantly lower in *Jdp2* KO Gabra6^+^ GCPs in the presence of NAC. In contrast, the level of Gabra6 positivity was about 1.7-fold higher in *Jdp2* KO vs. WT cells (Fig. S2B, C). Thus, Jdp2 might exert specific effects on Gabra6^+^ expression, but not on Gsk3β and NeuN-specific neural differentiation. Thus, we next focused on the triggering of the early commitment toward neural differentiation activity in *Jdp2*-depleted Gabra6^+^ GCPs, which indicated that Jdp2 might be a blocker of the NAC-mediated differentiation of Gabra6^+^ GCPs into the neural stages.

### Neuronal differentiation protocol of Gabra6^+^ GCPs

**Fig. 3:**
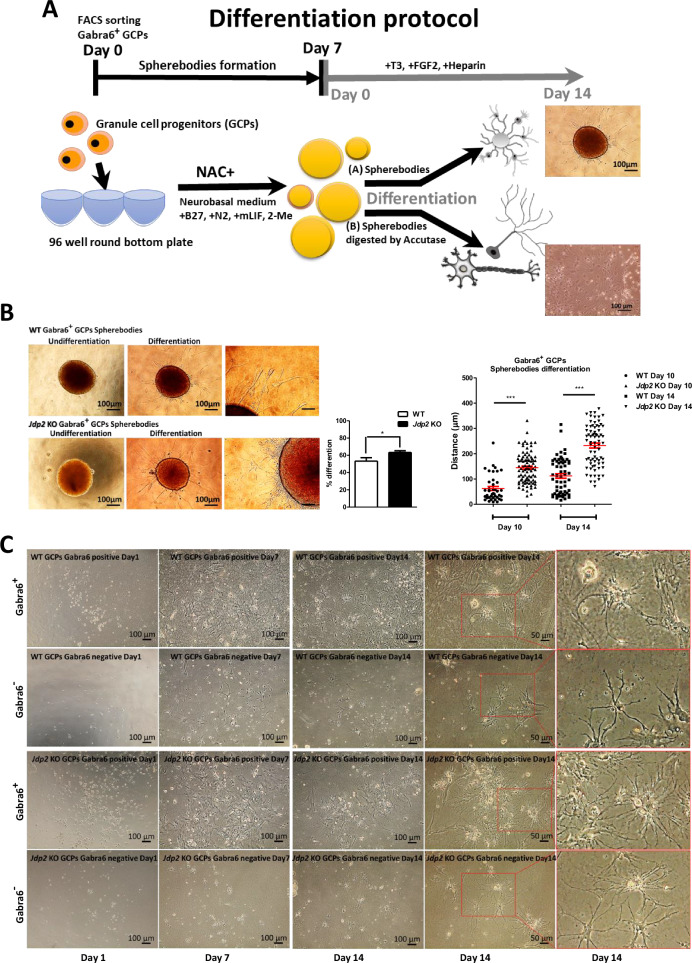
Differentiation of Gabra6^+^ GCPs into neural cells. **A** Two differentiation protocols of Gabra6^+^ GCPs were used: the sphere body method and the 2D-accutase culture method. A 96-well non-attached plate was used to generate sphere bodies, which were cultured in mouse GC medium supplemented with mLIF and 2-mercaptoethanol (2-ME) for 7 days. Then, the spherebodies were transferred to a differentiation medium containing brain-derived neurotrophic factor (BDNF), neurotrophin 3 (NT3), thyroid 3 (T3), and fibroblast growth factor 2 (FGF2), to induce Purkinje cells (PCs). The spherebodies were treated with accutase and plated in the 2D-plates and then committed to the differentiation as described in Materials and Methods. After 14 days, the differentiated cells were shown in respective panels. Scale bars: 100 and 50 μm. **B** Differentiation process of spherebodies derived from WT and *Jdp2* KO Gabra6^+^ GCPs. Undifferentiated GCP sphere bodies (left) and differentiated GCP sphere bodies (middle and right) are shown. Scale bars, 100 and 50 μm. The quantitative ratio of the sphere bodies differentiated from WT (*n* = 43) and *Jdp2* KO (*n* = 44) GCPs was measured. The neurite growth distance was measured on days 10 and 14. The *Jdp2* KO GCPs exhibited longer neurites from differentiated sphere bodies compared with WT GCP-derived neurites (*n* = 5: *P < 0.05, ***P < 0.001). **C** The differentiation of WT and *Jdp2* KO Gabra6^+^ GCPs was performed as described in the Materials and Methods. After digesting the respective sphere bodies with accutase, the cells were transferred into the differentiation medium and cultivated further for 1, 7, and 14 days. Two-dimensional (2D) cell cultivation from *Jdp2* KO Gabra6^+^ GCPs yielded a greater number of dendrites compared with WT Gabra6^+^ GCPs. After 14 days of differentiation, the bright-field images showed the presence of Purkinje cells (PCs). The Purkinje cells were stained using neuronal markers, for quantification, as shown in Fig. 4B. Scale bars, 100 and 50 μm.

**Fig. 4:**
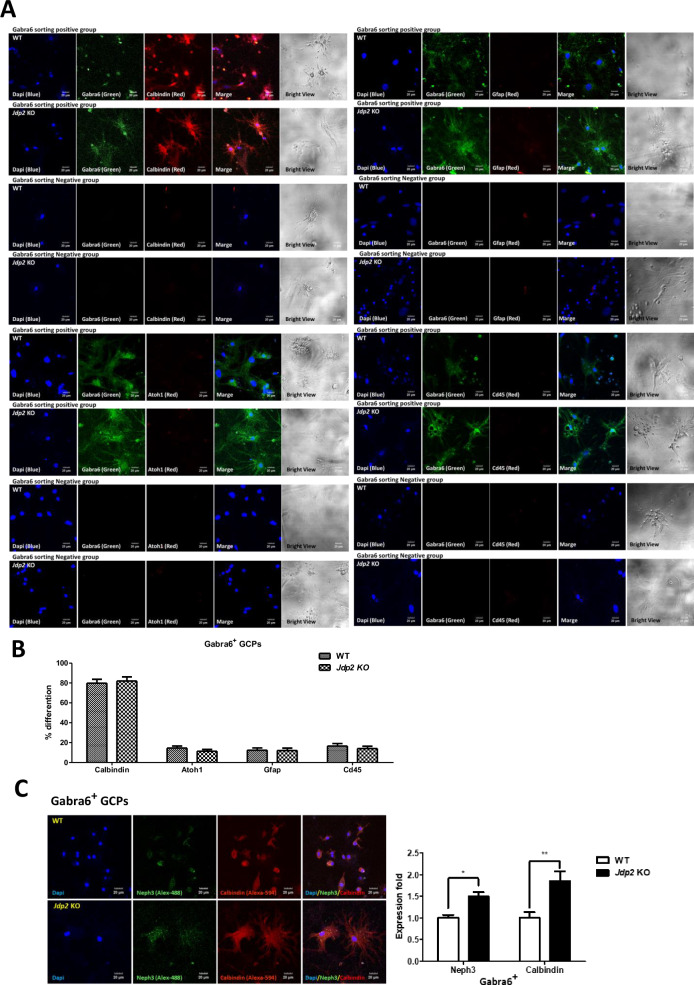
Comparison of expression of neuronal markers in Gabra6^+^ GCPs from WT and *Jdp2* KO mice after differentiation method of 2D cultivation-accutase treatment of the spherebodies for 14 days. **A** Gabra6^+^ GCPs from WT and *Jdp2* KO mice were generated the spherebodies for 7 days culture and digested with accutase and then cultured on 2D cells and then cultured for 14 days and examined the expression of differentiation markers on immunostaining. The different neurons were stained for the following marker proteins: Calbindin for Purkinje cells, Atoh1 for granule cells, GFAP for astrocytes, and CD45 for microglia. The blue color corresponds to DAPI staining, the green color corresponds to Gabra6 (as a control), and the neuronal markers were colored in red. We also present bright-field images. Scale bars, 20 μm. **B** In Gabra6^+^ GCPs sorted cell populations, the quantitation of different cell types was performed by counting double-positive signals for Gabra6 and neuronal markers. About 80% of the cell populations were Purkinje cells. The other cell types were less than 10% after differentiation for 14 days. **C** Immunostaining for Neph3 and calbindin in accutase-treated 2D-cultured *Jdp2* KO Gabra6^+^ GCPs and WT Gabra6^+^ GCPs after differentiation for 7 days. Scale bars, 20 μm. The expression of Neph3 and calbindin was compared between WT and *Jdp2* KO Gabra6^+^ GCPs. (*n* = 5; *P < 0.05; **P < 0.01).

Here, we developed two methods to achieve the neural differentiation of Gabra6^+^ GCPs into PCs. One method (A) consisted in the generation of sphere bodies in low-attached 96-well plates of Gabra6^+^ GCPs in neurobasal medium (B27 supplement, N2 supplement, mouse leukemia inhibitor factor (mLIF), and 2-mercaptoethanol) plus NAC for about 1 week, followed by commitment toward differentiation by replacing with differentiation medium (Fig. 3A) [12, 13]. After the incubation of Gabra6^+^-GCP-derived neurosphere bodies in the differentiation medium for 7 days, we found that the differentiation efficiency of *Jdp2* KO-derived neurosphere bodies was 1.2-fold faster than that of WT-derived neurosphere bodies (left panel, Fig. 3B). After further differentiation induction up to 10‒14 days, we found that the development of neurites in *Jdp2* KO Gabra6^+^ GCP-derived neurosphere bodies was better compared with that of neurites from WT Gabra6^+^ GCP-derived neurosphere bodies (right panels, Fig. 3B). The second method (B) consisted in the use of flat cultivation after digesting the sphere bodies with accutase, followed by re-cultivation for an additional 7–14 days. Specific neuronal fibers were clearly apparent after cultivation for an additional 7 days (Fig. 3C). More than 80% of the subpopulation of Gabra6^+^ GCPs was stained with anti-calbindin antibodies, whereas less than 8% of each population was positive for anti-Atoh1 (GC-specific), anti-GFAP (astrocyte-specific), and anti-CD45 (glia-specific) antibodies (Fig. 4A, B). This suggests that about 80% of Gabra6^+^ GCPs can differentiate into PCs in vitro in differentiation medium containing NAC.

To define the role of exposure to NAC in this differentiation process, we compared the expression of Purkinje progenitor cell markers, such as Neph3 [23, 24] and calbindin, between the condition of NAC exposure and that of the absence of NAC in Gabra6^+^ PGCs for 7 days. After NAC exposure of Gabra6^+^ PGCs prepared using an accutase-2D cell method, we found that PC makers, such as calbindin 3 and Neph3, were predominant (about 1.8-fold- and 1.5-fold-higher expression, respectively) in *Jdp2* KO vs. WT cells after 2 weeks of the neural differentiation protocol (Fig. 4C). These results suggest that Jdp2 controls the frequency and the speed of differentiation of GCPs into PCs. Therefore, the Jdp2 protein itself can prevent the neural differentiation of Gabra6^+^ GCPs into Purkinje neurons.

### Enhancement of Ca^2+^ signals in *Jdp2* KO Gabra6^+^ GCPs

**Fig. 5:**
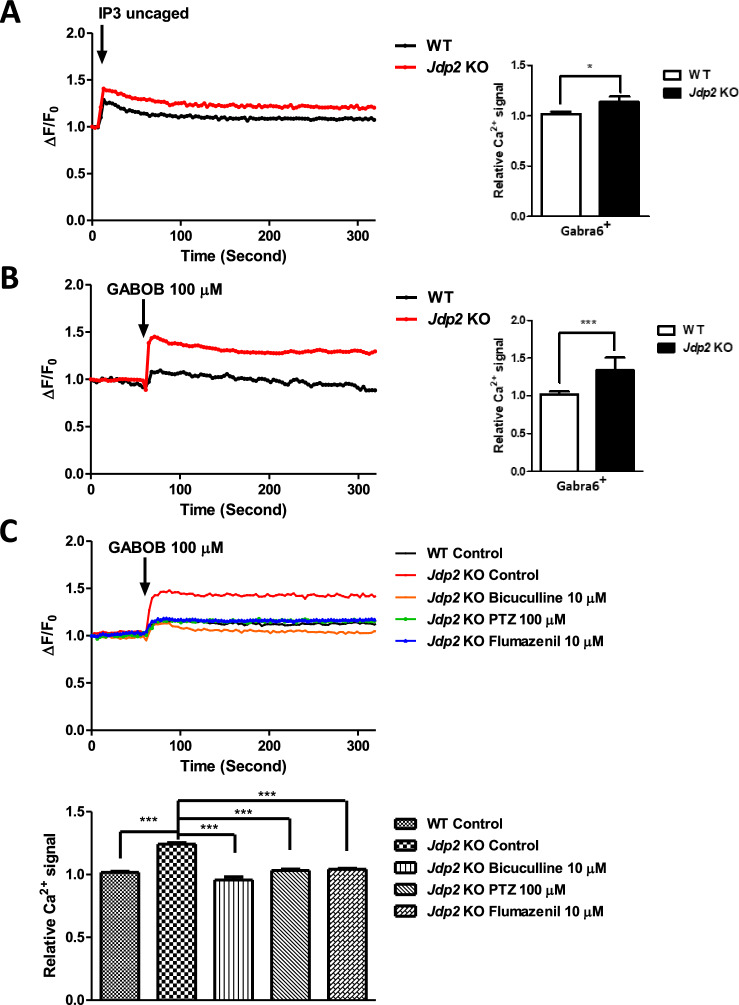
Calcium uptake studies in accutase-treated 2D-cultured cells on day 7 of differentiation. **A** Calcium-uptake activities of WT and *Jdp2* KO Gabra6^+^ GCPs. Inositol triphosphate (IP3)-mediated Ca^2+^ liberation was assessed in neuronal cells using a two-beam-signal cell simulation system. The IP3 (0.5 μM) stimulation ΔF/F ratio and the calcium-uptake activities were evoked to a higher degree in *Jdp2* KO GCPs after IP3 uncaging (*n* = 5; *P < 0.05). **B** A Gaba receptor agonist (GABOB) (100 μM) stimulated a greater release of Ca^2+^ in *Jdp2* KO Gabra6^+^ GCPs (1.34^-^fold higher compared with WT GCPs) (*n* = 5; ***P < 0.001). **C** The effects of Gaba receptor inhibitors, i.e., bicuculline (10 μM), PTZ (100 μM), and flumazenil (10 μM), on GABOB stimulated the release of Ca^2+^ in WT and *Jdp2* KO Gabra6^+^ GCPs. The protocol used for the liberation of Ca^2+^ was as described in the Materials and Methods (*n* = 5; *P < 0.05; **P < 0.01; ***P < 0.001).

A previous study demonstrated that the Gaba receptor is involved in the regulation of the differentiation of rodent neural progenitor cells [23]. The Gaba receptor is a G-protein-coupled receptor that is associated with inositol 1,4,5-triphosphate (IP3)-induced Ca^2+^ signals [25–27]. We hypothesized that the Gaba-receptor-mediated Ca^2+^ signals are involved in *Jdp2*-regulated neural differentiation. To investigate the effect of *Jdp2* on Gaba-receptor-mediated Ca^2+^ signals, we examined the caged inositol IP3-mediated Ca^2+^ uptake, which leads to local calcium release, in accutase-2D-differentiated Gabra6^+^ GCPs (Fig. 5). In the case of calcium release in *Jdp2* KO cells, the level of uncaged IP3 was 1.1-fold higher than that detected in WT cells (Fig. 5A). This uncaged-IP3-mediated Ca^2+^ uptake was modulated by the Gaba receptor. Thus, to confirm the Gaba-receptor-regulated IP3-mediated calcium release, the Gaba receptor agonist GABOB was added to the culture medium. The calcium uptake in *Jdp2* KO accutase-2D-differentiated Gabra6^+^ GCPs was 1.3-fold higher than that observed in WT cells after GABOB stimulation (Fig. 5B). Taken together, these results suggest that the ability to trigger calcium signaling is evoked by a higher calcium uptake in *Jdp2*-depleted accutase-2D-differentiated Gabra6^+^ GCPs via intracellular uncaging. To examine the role of the Gaba receptor in inositol IP3-mediated Ca^2+^ uptake in *Jdp2* KO accutase-2D-differentiated Gabra6^+^ GCPs, we used various Gaba receptor inhibitors, such as bicuculline, PTZ, and flumazenil **(**Fig. 5C). The calcium uptake in *Jdp2* KO accutase-2D-differentiated Gabra6^+^ GCPs after GABOB stimulation was reversed to the original levels detected in control GCPs.

### Expression of PC-related and Gaba-receptor-related genes

**Fig. 6:**
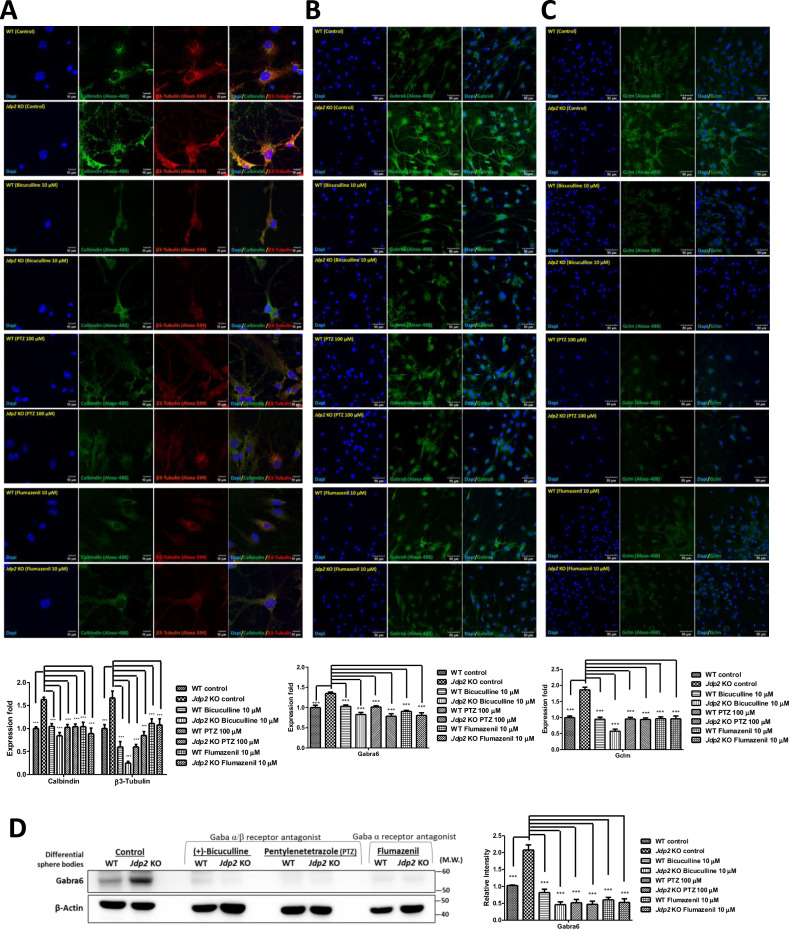
Gaba receptor antagonists block the neuronal differentiation of Gabra6^+^ GCPs from *Jdp2* KO mice to PCs. Immunostaining of accutase-treated 2D cultures of WT and *Jdp2* KO Gabra6^+^ GCPs for 7 days in the presence of NAC and neurites after induction toward neural differentiation via Gaba receptor inhibitors, i.e., bicuculline (10 μM), PTZ (100 μM), and flumazenil (10 μM), using antibodies against calbindin and tubulin βIII (**A**), Gabra6 (**B**), and Gclm (**C**). The staining was performed as described in the Materials and Methods. The nucleus was stained with DAPI. Scale bars, 10 μm. The expression of each protein in WT Gcbra6^+^ GCPs was set as 1.0 (*n* = 3; ***P < 0.001). **D** Western blot analysis of the expression of Gabra6 in accutase-treated 2D-cultivated *Jdp2* KO vs. WT Gabra6^+^ GCPs for 7 days in the presence of NAC and in the presence of inhibitors, i.e., bicuculline (10 μM), PTZ (100 μM), and flumazenil (10 μM). The expression of each protein in WT Gabra6^+^ GCPs was set as 1.0 (*n* = 3, ***P < 0.001). The relative value was normalized to β-actin and is presented as a ratio. Uncropped raw data was shown in Fig. S5.

To examine the role of the Gabra6, we used various GABA receptor inhibitors [28], such as bicuculline and PTZ (specific for α/β), and flumazenil (a competitive antagonist at the benzodiazepine-binding site on α) and assessed whether they inhibited neural differentiation (Fig. 6A–D). These inhibitors blocked the expression of calbindin and βIII tubulin (Fig. 6A), Gabra6 (Fig. 6B), and the glutamate–cysteine ligase modifier subunit (Gclm, known as gamma-glutamyl cysteine; glycine ligase or glutathione synthetase) [29] (Fig. 6C). These results indicate that the Gabra6 plays a critical role in NAC-mediated antioxidation, for the accumulation of GSH, by increasing the glutamine–cysteine pump including Gclm, and determines the neural network in *Jdp2*-depleted Gabra6^+^ GCPs, including PC neurons.

In an attempt to search for mutations in the *GABRA6, GABRA1, GABRB2* genes, as well as the antioxidative gene *NRF2*, cysteine transporters (such as *SLC7A11* and *CD44v*), and their regulators (such as *JDP2* and *p21*^*Cip1*^ (*CDKN1A*) in brain tumors, the cBioPortal (http://www.cbioportal.org/faq#how-do-i-cite-the-cbioportal) data were accessed. In total, 8139 patients and 8597 samples from 26 studies were grouped for each item, such as signaling, apoptosis, and cell-cycle-progression pathways [30–32]. The frequency of mutation in the *GABRA6*, *GABRA1*, and *GABRB2* genes was significantly higher than that detected for other cell-cycle- and cell-proliferation-related genes; furthermore, their co-occurrence in patients with cancer was examined. Thus, alterations in the *GABRA* gene family seem to be closely related to cancer occurrence, not only in mice, but also in human patients with brain cancer, through the regulation of the antioxidative response genes to produce GSH via SLA7A11 and CD44v, as an xCT receptor. A synthetic analogue of GABA was reported to play roles in anti-inflammation and as an antioxidant in mice [33] and pigs [28]. As shown in Figs S3A–C and S4A, B, the mutation rates of GABA receptors were significantly increased [30–32]. Accordingly, molecules that control the antioxidation response, such as GSH production-related molecules (e.g., SLA7A11, CD44v, p21^Cip1^ (CDKN1A), and JDP2), were also mutated significantly by gene amplification and deep deletions, similar to the gene mutation events observed in the GABA receptor family. GABRA1 might be related to NRF2 and CD44v, GABRA2 is related to CD44v, and GABRA6 is related to SLC7a11 (Figs. S3 and S4). These databases suggest that JDP2 is a possible regulator of the GABA receptor family signaling which might be concerned with ROS-antioxidation balance and neural differentiation from PGCs to PCs.

## Discussion

We developed a critical in vitro culture condition for mouse Jdp2-depleted Gabra6^+^ GCPs in the presence of NAC that allowed the trans-differentiation capacity to PCs. Because NAC is frequently used as an antioxidant [34–37], we tested the level of total glutathione (GSH) and ROS in this context. In general, NAC upregulates GSH and downregulates ROS in GCPs. We focused on the markers of brain stemness and differentiation at an early stage [38]. The Lgr5 stemness markers and Sox2/Klf4 were highly expressed in *Jdp2* KO compared with WT cells (Fig. 1D, F). In addition, the expression of antioxidation-related factors, such as Nrf2 and p21^Cip1^, was increased, whereas that of AhR was decreased in *Jdp2* KO vs. WT cells (Fig. 1E). This might be important to show the difference between these stemness and antioxidation markers in brain Gabra6^+^ GCPs with NAC in the case of trans-differentiation. The BrdU-incorporation experiment and cell-cycle analysis indicated that the G_2_/M ratio in Gabra6^+^-*Jdp2* KO GCPs was lower than that observed in WT cells (Fig. 2A–C). In fact, during the differentiation-commitment stages, DNA synthesis was decreased, and apoptosis was increased in *Jdp2* KO Gabra6^+^ GCPs. Moreover, the cells seemed to enter differentiation. Treatment with NAC enhanced this stage in Gabra6^+^ GCPs collected from the cerebella of *Jdp2* KO mice, which might affect the antioxidation control.

We successfully generated a primary culture system of PGCs in the presence of NAC for more than 2 weeks in vitro. We also established a protocol for the generation of sphere bodies after 1 week of culture, and maintained the stemness of GCPs by adding mLIF, to avoid cell differentiation. Subsequently, the medium was changed to a differentiation medium that included T3, FGF2, and heparin. The proliferation of Gabra6^+^ GCPs from WT mice was greater than that of cells from *Jdp2* KO mice. However, in the differentiation experiment, the number of neurosphere bodies obtained from *Jdp2* KO mice was higher than that obtained from WT-derived neurosphere bodies; moreover, the onset of differentiation in *Jdp2* KO cells was also higher than that of WT Gabra6^+^ GCPs after measuring the neurite growth from neurosphere bodies (Fig. 3B). In the case of pre-differentiation, the *Jdp2* KO spheres committed to differentiation toward the neural cascade compared with WT spheres. Furthermore, we found that staining signals for PC-specific biomarkers were clearly detected during the differentiation stage. Moreover, a significantly higher level of expression of calbindin was detected in *Jdp2* KO compared with WT Gabra6^+^ GCPs (Fig. 4B, C). The comparison of the differentiation frequency between the standard cultivation method with NAC and the in vitro differentiation culture condition revealed that the expression of calbindin in the differentiation medium in *Jdp2* KO cells was higher compared with that observed for WT Gabra6^+^ GCPs; however, we detected a significant trans-differentiation of Gabra6^+^ GCPs to PCs, even in the NAC culture condition (Fig. 4C).

**Fig. 7:**
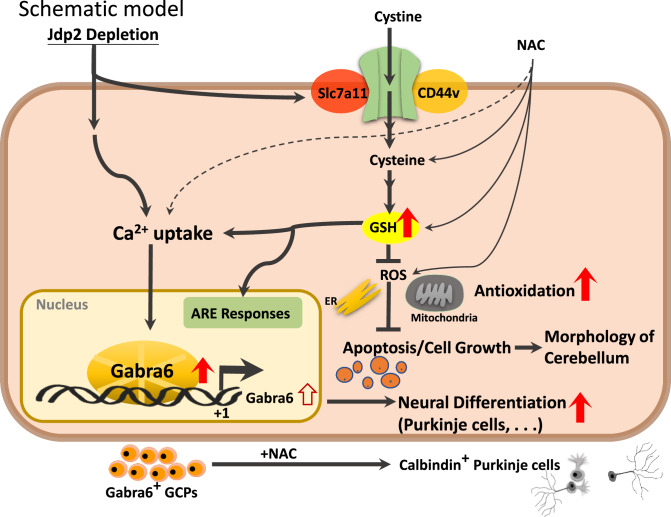
Hypothetical model of the effect of *Jdp2* depletion and Gaba receptor-family and antioxidation response in the initiation of neural differentiation. Schematic models of the pathways of *Jdp2* depletion in the presence of NAC for the neural differentiation of GCPs. NAC induces xCT channels and calcium uptake, to generate antioxidation, including GSH production, thus inducing *GAabra1* and *6* gene activation, which might result in the neural induction of PGCs. The original of the figure presented in Fig. S4 was by C–C. Ku et al. [10, 11], with permission to reuse and modify.

Previously, it was reported that calcium ions are among the most critical signaling molecules because they control almost all cellular functions and processes [25, 39]. Changes in intracellular free calcium concentrations are closely correlated with the signal transduction of G-protein-coupled receptors and various cellular pathophysiological conditions, such as spinocerebellar ataxia, PD, Gillespie syndrome, and HD [25–27, 40]. Therefore, the measurement of free intracellular calcium is critical for understanding calcium-dependent neuronal activity. As depicted in Fig. 5, the depletion of Jdp2 caused a higher Gaba receptor-IP3-mediated calcium release compared with that observed in WT GCPs. The expression levels of the Gaba receptor and Purkinje markers, as well as IP3-mediated calcium, were closely related to the function of Jdp2 depletion in the presence of NAC. In turn, the Gaba receptor antagonists bicuculline, PTZ, and flumazenil inhibited the differentiation of GCPs to neurons, the levels of calbindin (Fig. 6A). Gabra/Gabrb antagonists and a Gabra antagonist inhibited the expression of Gabra6 and Gclm (Fig. 6B, C). These data indicate that a strong correlation between Gabra and PCs is critical for the developmental control of Purkinje cell dendrites and tumor phenotypes [39, 41]. In addition, the inhibitory synapses that occur during neural development are regulated by GABAergic synaptic lateral diffusion dynamics, which are tuned by Ca^2+^ and glutamate [27]. In fact, the marker genes of the GABRA receptor families and cystine transporter xCT (SLA7A11 and CD44v), as well as antioxidation-controlled factors, such as JDP2, NRF2, and CDKN1A (p21^Cip1^), were significantly mutated in brain tumors, e.g., glioblastoma, glioma, and neuroblastoma (Fig. S4A, B). Taken together, our results suggest that Jdp2 is critical for inhibiting the normal differentiation of GCPs into functional PCs through an IP3-mediated calcium-uptake function via the Gaba receptor alpha 6 (Fig. 7). Further molecular studies are required to understand the Jdp2-mediated IP3 generation axis, Gaba receptor function, and calcium uptake, which were required here for the neural differentiation of GCPs into PCs. In addition, Jdp2 might be a key regulator of the suppression of the cell cycle progression and, eventually, of the differentiation program of Gabra6^+^ GCPs into PCs. Thus, the understanding of how Jdp2 controls the cell cycle and the differentiation of Gabra6^+^ GCPs into functional PCs through calcium uptake is critical for the development of the use of Jdp2 to treat PC-mediated genetic diseases and related behavioral disorders.

In summary, the present study demonstrated first time that the trans-differentiation of Gabra6^+^ GCPs toward neuron lineages, especially PCs, was triggered by their exposure to the NAC, to induce antioxidation signaling, including calcium uptake and the activation of the Gabra6. The deletion of Jdp2 also activated the early differentiation program of Gabra6^+^ GCPs in vitro. Thus, mutation and deletion of the Jdp2 molecule in the urgent stress might trigger this determination of Gabra6^+^ GCPs toward neurons, including Purkinje neurons, after exposure to NAC. Therefore, we speculate that antioxidant drugs might be effective agents for rescuing the oxidative-stress-induced Gabra6^+^ GCP damages that occur in the absence of a normal functional Jdp2 in the cerebellum.

## Supplementary information

Supplementary material file PDF

Supplementary Figure 5, uncropped Western blots

## Data Availability

All studies are included in the article and/or datasets S1–S4 and S5 (uncropped Western blots).
