# Supplementary material for: Trans-differentiation of Jdp2-depleted Gaba-receptor-positive cerebellar granule cells to Purkinje cells
Source: Cell Death Discov. 2024 Dec 18;10:500. doi: 10.1038/s41420-024-02262-2 (PMC11655981; doi:10.1038/s41420-024-02262-2)
Supplement: Supplementary file 1 — Supplementary material file PDF [file 41420_2024_2262_MOESM1_ESM.pdf]

Supplementary materials

Trans-differentiation of Jdp2-depleted Gaba-receptor-positive cerebellar granule cells to Purkinje cells

Chia-Chen Ku[1-3], Jia-Bin Pan[1-,3], Kenly Wuputra[1-3], Wen-Li Hsu[2,4,14], Kohsuke Kato[5], Michiya Noguchi[6], Yukio Nakamura[6], Shigeo Saito[7], Cheng-Yu Tsai[1,8-10], Ying-Chu Lin[11], Deng-Chyang Wu[2,12]*, Chang-Shen Lin[1,13], and Kazunari K. Yokoyama[1-3]*

[1]Graduate Institute of Medicine, Kaohsiung Medical University, Kaohsiung 80708, Taiwan,

[2]Regenerative Medicine and Cell Therapy Research Center, Kaohsiung Medical University, Kaohsiung 80708, Taiwan.

[3]Cell Therapy and Research Center, Kaohsiung Medical University Hospital, Kaohsiung 80756, Taiwan.

[4]Department of Dermatology, Kaohsiung Municipal Ta-Tung Hospital, Kaohsiung Medical University Hospital, Kaohsiung Medical University, Kaohsiung 80145, Taiwan.

[5]Department of Infection Biology, Graduate School of Comprehensive Human Sciences, the University of Tsukuba, Tsukuba 305-8577, Japan.

[6]Cell Engineering Division, RIKEN BioResource Research Center, Tsukuba. Ibaraki 305-0074, Japan.

[7]Saito Laboratory of Cell Technology, Yaita 329-1571, Tochigi, Japan.

[8]Division of Neurosurgery, Department of Surgery, Kaohsiung Medical University Hospital, Kaohsiung 80756, Taiwan.

[9]Division of Neurosurgery, Department of Surgery, Kaohsiung Medical University Gangshan Hospital, Kaohsiung 820, Taiwan.

[10]Department of Post-Baccalaureate Medicine, Kaohsiung Medical University, Kaohsiung 80708, Taiwan.

[11]School of Dentistry, Kaohsiung Medical University, Kaohsiung 80708, Taiwan,

[12]Division of Gastroenterology, Department of Internal Medicine, Kaohsiung Medical University Hospital, Kaohsiung 820, Taiwan.

[13]Department of Biological Sciences, National Sun Yat-sen University, Kaohsiung 80424, Taiwan.

[14]Present address: National Center for Geriatrics and Welfare Research, National Health Research Institutes, Yunlin County 632007, Taiwan.

*To whom correspondence may be addressed; Kazunari K. Yokoyama (kazu@kmu.edu.tw; Cell Therapy and Research Center, Kaohsiung Medical University Hospital; Graduate Institute of Medicine, Kaohsiung Medical University, Taiwan, Tel; +886-7312. 1001, ext 2729; FAX; +886-7313-3849), and Deng-Chyang Wu (dechwu@kmu.edu.tw; Division of Gastroenterology, Department of Internal Medicine, Kaohsiung Medical University Hospital, Taiwan). ORCID of KK. Yokoyama; 0000-0001-8508-7587; CS Lin; 0000-0001-7415-2187; K. Wuputra; 0000-0003-4026-7052; WL Hsu; 0000-0003-1034-4977; YC Lin; 0000-0002-2499-8632, and CC Ku; 0000-0002-1496-3081.

Supplementary Materials

1. **Materials and Methods**
2. **Supplementary legends of Figures 1-5**
3. **Supplementary Tables 1-2**

MATERIALS AND METHODS

Animals and cells

The animal welfare guidelines for the care and use of laboratory animals used here were those published by the RIKEN BioResource Research Center (BRC) in Japan (Kiteisv.intra.riken.jp/JoureiV5HTMLContents/act/print/print110000514.htm), the Animal Care Committee of the National Laboratory Animal Center (NLAC) (106022), and Kaohsiung Medical University in Taiwan (106189; 107128; 108244). All animal experiments were performed in accordance with these approved guidelines. The strategy used to generate the *Jdp2* KO mouse model was described previously [1–5].

Primary culture of GCPs

The preparation of GCPs from newborn mice (postnatal days 5–7) was carried out as described previously [1, 2, 6, 7]. In brief, the meninges and skull were excluded, and the cerebellum was stored in cold Hanks–glucose buffer (Thermo Fisher Scientific, Waltham, MA, USA). To digest the single cells from cerebellar tissues, we used Earle’s balanced salt solution containing papain (20 U/ml; Worthington, Lakewood, NJ, USA), 1 mM L-cysteine, 0.5 mM ethylene diamine tetra-acetic acid, and 0.05% DNase1 at 37 °C for 15 min [1, 2]. The tissues were pipetted repeatedly to obtain a suspension of single cells, followed by centrifugation at 200 × *g* for 5 min. After discontinuous density gradient centrifugation, the cell pellets were resuspended in 3 ml of EBSS, and then 5 ml of EBSS containing 0.2% ovomucoid and a 0.1% bovine serum albumin (BSA) inhibitor solution were then added carefully onto the upper layer, followed by centrifugation at 70 × *g* for 6 min [8]. The dishes and plates were pre-coated in 50 μg/ml poly-D-lysine (BD

Biosciences, San Jones, CA, USA) using the following culture medium: neurobasal A medium, 2% B-27 supplement, 100 U/ml penicillin, 100 μg/ml streptomycin, 2 mM glutamine, 5 μg/ml insulin, 100 μg/ml apo transferrin, and 16 μg/ml putrescine (all from Thermo Fisher Scientific, Waltham, MA, USA); plus 1 mM sodium pyruvate, 100 μg/ml BSA, 30 μM N-acetyl cysteine, 62 ng/ml progesterone, 40 ng/ml sodium selenite, and 5 ng/ml epidermal growth factor (all from Merck KGaA, Darmstadt, Germany).

Fluorescence-activated cell sorting (FACS) of GCPs using anti-Gabra6 antibodies

FACS using GCPs was carried out to separate the subpopulation based on Gabra6–FITC labeling. The GCPs were washed with 10 mM Dulbecco's modified phosphate basal saline (PBS) and detached using trypsin (Thermo Fisher Scientific) in the presence of DNase (Merck). Trypsinization was stopped using 10% fetal bovine serum and the cells were washed with PBS containing 0.5% BSA. Subsequently, the cells were centrifuged at 1,000 rpm for 5 min at 4 °C and resuspended in 300 μl of PBS containing 0.5% BSA. The PGCs were cultivated in the presence of 30 μM NAC for 7 days to commit the neural differentiation. Then, the cells were labeled with Gabra6–FITC antibodies (1:100; Bioss, Woburn, MA, USA, bs-12063R-FITC), which were added to labeled GCPs, followed by a 30-min incubation. Finally, GCPs labeled with Gabra6–FITC antibodies were washed by PBS containing 0.5% BSA, and the samples were then analyzed on a BD FACSMelody™ Cell Sorter (BD Biosciences, New Jersey, USA), to sort each subpopulation. The data were plotted and quantified using FlowJo (v. 10; BD Biosciences). The sorted Gabra6-positive GCPs (Gabra6$^+$ GCPs) and Gabra6-negative GCPs (GAabra6$^-$ GCPs) were cultured as described above.

Incorporation of bromodeoxyuridine (BrdU)

BrdU Cell Proliferation Assay Kits (Cell Signaling Technology, Beverly, MA, USA) were used to detect the sorted GCPs, according to the manufacturer's instructions. For immunocytochemistry of the stained cells, fixed GCs stained with the anti-BrdU antibody were developed using streptavidin–HRP plus ChemMate™ DAKO EnVision™ Detection kits (Dako, Glostrup, Denmark; K5007). The cells were visualized using an Olympus CKX41 microscope (Olympus, Tokyo, Japan), and the resulting images were quantified using the open access Fiji/ImageJ analytical software (https://imagej.net/Fiji) [9].

Immunocytochemistry

GCPs or Gabra6$^+$ GCPs/Gabra6$^-$ GCPs were subjected to immunocytochemistry before or after cultivation as described elsewhere [1, 2]. The primary and secondary antibodies were shown in Supplemental Table 1. Cells were mounted on slides using the ProLong® Gold antifade mounting medium (Molecular Probes, Thermo Fisher Scientific; P36934), and cell immunofluorescence was visualized using a FV1000 confocal laser scanning microscope (Olympus, Tokyo, Japan), and the resulting images were quantitated using the open access Fuji/ImageJ analytical software (https://imagej.net/Fuji) [9].

Differentiation of Gabra6$^+$-GCPs into PCs

Method A; Formation of sphere bodies via GCP aggregation; The FACS sorted GCPs by anti-Gabra6 antibodies were cultivated in the presence of NAC to aggregate neurosphere bodies in low-attachment 96-well plates. The culture conditions were the same as those described above for the GC primary culture, with addition of the mouse leukemia inhibitory factor (mLIF; Millipore, Darmstadt, Germany) and 2-

mercaptoethanol (2-Me; Merck, 10 nM).

Method B; Two dimension (2D) flat cell culture after digesting by accutase; The sphere bodies of Gabra6^{+}-GCPs were cultivated for 7 days in the presence of NAC and then these developing sphere bodies were digested by Accutase (Merk, SCR 005) as described in the manufacturer's protocols. The differentiation medium was consisted of the growth-factor-free chemically defined medium (gfCDM) [10] containing insulin [11] termed ESF5 medium [2], was used as a differentiation condition medium and included insulin (10 μg/ml), transferrin (5 μg/ml), 2-aminoethanol (10 μM), 2-mercaptoethanol (10 μM), and sodium selenite (20 nM) (all from Merck). Several recombinant proteins, i.e., FAF-BSA (100 μg/ml; Merck, A8806-5G), the brain-derived neural factor (BDNF) (50 ng/ml; ProSpec, Ness-Ziona, Israel, cyt-688-b), NT3 (50 ng/ml; ProSpec, cyt-919-b), T3 (10 ng/ml; Sigma-Aldrich, Merk, T5516-1MG)[50], FGF2 (10 ng/ml; R&D Systems Inc., Minneapolis, MN) [2], and heparin (100 ng/ml; Merck, H3149-10KU), were also used for the induction of differentiation.

Western blotting

Western blotting experiments were performed as described previously [1, 2]. The PVDF membranes were then probed with primary antibodies against CACNα1A (1:1000; MyBioSource, mbs176970), calbindin (1:1000; Cell Signaling Technology, 13176), GABRα1 (1:1000; R&D, PPS022), GABRα6 (1:1000; Bioss, BS12063R), GABRβ2 (1:1000; R&D, PPS031), GRIN2A (1:1000; R&D; PPS012), PCP4 (1:1000; MyBioSource, mbs2517367), and VGLUT1 (1:1000; Cell Signaling Technology, 12331); followed by incubation with the secondary antibodies: anti-rabbit IgG HRP-conjugated antibody (1:3000; Cell Signaling Technology, 7074) and anti-mouse IgG HRP-conjugated

antibody (1:3000; Cell Signaling Technology, 7076). The results were analyzed using a ChemiDoc XRSPlus instrument (Bio-Rad, Hercules, CA, USA).

Immunocytochemistry

GCPs or Gabra6^{+} GCPs/Gabra6^{-} GCPs were subjected to immunocytochemistry before or after cultivation as described elsewhere [1, 2]. The primary antibodies were against Atoh1 (1:200; Chemicon, AB5692), Neph3 (1:200; MyBioSource, mbs153480), calbindin (1:200; Cell Signaling Technology, 13176), Gabra6 (1:200; Bioss, BS12063R), P/Q-type (1:200; Santa Cruz, sc-390004), and β3-tubulin (1:200; Cell Signaling Technology, 4466). After washing with PBST, the cells were incubated for 1.5 h with the following secondary antibodies: Alexa Fluor® 594-conjugated goat anti-rabbit IgG (Life Technologies, Grand Island, NY, USA; A11037), Alexa Fluor® 594-conjugated goat anti-mouse IgG (Life Technologies; A11032), and Alexa Fluor® 488-conjugated goat anti-rabbit IgG (Life Technologies; A11034); then processed using 4′,6-diamino-2-phenylindole (DAPI), to visualize cell nuclei (1:3000; Merck). Cells were mounted on slides using the ProLong® Gold antifade mounting medium (Molecular Probes, Thermo Fisher Scientific; P36934), and cell immunofluorescence was visualized using a FV1000 confocal laser scanning microscope (Olympus, Tokyo, Japan).

Formation of neurosphere bodies via GCP aggregation

In the case of differentiation, primary culture of sorted GCPs was performed to aggregate neurosphere bodies in low-attachment 96-well plates. The culture conditions were the same as those described above for the GC primary culture, with addition of the mouse leukemia inhibitory factor (mLIF; Millipore-Merck) and 2-mercaptoethanol (10 nM,

Merck, W7522), to maintain the stemness of GCPs and inhibit cell differentiation.

RNA sequencing, gene clustering, and gene categorization

RNA sequencing was conducted using a Genome Analyzer IIX System (Illumina, San Diego, CA, USA) according to the 50-bp single-end protocol by Welgene Biotech (Taipei, Taiwan), as described previously [1, 2]. The sequences obtained were subjected to a filtering process using ConDeTri [13], to obtain qualified reads, which were investigated and estimated by TopHat/Cuffdiff [14]. The Human Genome Build 19 and gene features were retrieved from the Ensemble database and used for processing. The gene expression levels were calculated as reads/kilobase of transcript/million mapped reads (RPKM). Differentially expressed genes were filtered using an RPKM ≥0.3, a fold change ≥2, and a P value < 0.05. RNA sequencing data were deposited in the NCBI Bioproject Database (http://www.ncbi.nlm.nih.gov/bioproject) with the accession numbers SUB3541857, SUB3541902, SUB3541913, and SUB3541945.

Hierarchical clustering of the genes was performed as follows. First, gene-level normalization was performed by transforming the RPKM of each gene of each sample to a Log2 median-centered ratio. Subsequently, clustering was obtained using Euclidean distance and complete linkage settings. Finally, a heatmap was generated by coloring each gene on the Log2 median-centered ratio. To convert gene symbols to Ensemble gene accessions, the unique gene symbols of each topic were mapped to Ensemble official symbols.

Cancer genome atlas/cBioPortal analysis

Large-scale cancer genomics projects such as The Cancer Genome Atlas [15] are

generating an overwhelming amount of cancer genome data from different technical platforms. The cBioPortal for Cancer Genomics (http://cbioportal.org) provides a Web resource for exploring, visualizing, and analyzing multidimensional cancer genomics data. The portal reduces molecular profiling data from cancer tissues and cell lines into readily understandable genetic, epigenetic, gene expression, and proteomic events [16, 17]. This accelerates the translation of genomics data into the identification of cascades, therapies, and clinical trials. The cBioPortal (http://www.cbioportal.org/faq#how-do-i-cite-the-cbioportal) data were accessed (Sept. 21st, 2022), and we surveyed gene mutation maps of GABA receptors, xCT channels, GSH, antioxidation response-related proteins. Finally, 8,139 patients and 8,597 samples from 26 studies were summarized in the cBioPortal for Cancer Genomics [16, 17] in brain tumors.

Statistical analysis

Data are presented as the mean ± standard error of the mean. Statistical comparisons between experimental conditions were conducted using GraphPad Prism 5.0 (GraphPad Software, San Diego, CA, USA). For multiple comparisons, a one-way analysis of variance (ANOVA) followed by Tukey's test were performed using GraphPad Prism 7.0 (GraphPad, San Diego, CA, USA). An unpaired, two-tailed Student's *t*-test was used to compare the control and treatment groups. The Mann–Whitney nonparametric median statistical test was used for analyses of cell areas. All differences were designated as being statistically significant at $P < 0.05$.

References

1. Ku CC, Wuputra K, Kato K, Pan JB, Li CP, Tsai MH, et al. Deletion of Jdp2 enhances Slc7a11 expression in Atoh-1 positive cerebellum granule cell progenitors in vivo. Stem Cell Res Ther 2021; 12: 369.
2. Ku CC, Wuputra K, Kato K, Lin WH, Pan JB, Tsai SC, et al. Jdp2-deficient granule cell progenitors in the cerebellum are resistant to ROS-mediated apoptosis through xCT/Slc7a11 activation. Sci Rep 2020; 10; 4933.
3. Pan J, Nakade K, Huang YC, Zhu ZW, Masuzaki S, Hasegawa H, et al. Suppression of cell-cycle progression by Jun dimerization protein-2 (JDP2) involves downregulation of cyclin-A2. *Oncogene* 2010, 29: 6245-6256.
4. Nakade K, Pan J, Yoshiki A, Ugai H, Kimura M, Liu B, et al. JDP2 suppresses adipocyte differentiation by regulating histone acetylation. *Cell Death Differ* 2007, 14: 1398-1405.
5. Tanigawa S, Lee CH, Lin CS, Ku CC, Hasegawa H, Qin S, et al. Jun dimerization protein 2 is a critical component of the Nrf2/MafK complex regulating the response to ROS homeostasis. *Cell Death & Disease* 2013, 4: e921-e921.
6. Lee HY, Greene LA, Maspn CA, Manzini MC. Isolation and culture of post-natal mouse cerebellar neurn progenitor cells and neurons. *J Vis Exp* 2009, 12: 990.
7. Malach R. Coryical Column as devices for maxizing neura; diveresity. *Trends Neurosci* 1994, 17: 101-104.
8. Kawaguchi K, Habara T, Terashima T, Kikkawa S. GABA modulates development of cerebellar Purkinje cell dendrites under control of endocannabinoid signaling. *J Neurochem. 2010, 114:627-638.*
9. Rueden CT, Scgindelin J, Hiner, MC, DeZonia, BE, Waletr AE, Arena ET, et al. ImageL2: Image J for the bext generation of scientific imnage data. *BMC Bioinofmraitrcs* 2017, 18: 529.
10. Wataya T, Ando S, Muguruma K, Ikeda H, Watanabe K, Eiraku M, et al. Nininization of exogenous signals in ES culture induces rostral hypothalmic differemntiation. *Proc Natl Acda Sci USA* 2008, 105: 11796-11801.
11. Yamasaki S, Nabeshima K, Sotomaru Y, Taguchi Y, Mukasa H, Furue MK, et al. Long-term serial cultivation of mouse induced pluripotent stem cells in serum-free and feeder-free defined medium. *The International journal of developmental biology* 2013, 57: 715-724.
12. Hatsukano T, Kurisu J, Fukumitsu K, Fujishima K, Kengaku M. Thyroid Hormone Induces PGC-1α during Dendritic Outgrowth in Mouse Cerebellar Purkinje Cells. *Front Cell Neurosci* 2017, 11: 133 (2017).
13. Smeds L, Künstner A. ConDeTri--a content dependent read trimmer for Illumina data. *Plos one* 2011, 6: e26314.
14. Trapnell C, Roberts A, Goff L, Pertea G, Kim D, Kelley DR, et al. Differential gene and transcript expression analysis of RNA-seq experiments with TopHat and Cufflinks. *Nature protocols* 2012, **7**: 562-578.
15. Ciriello G, Miller ML, Aksoy BA, Senbabaoglu Y, Schultz N, Sander C. Emerging landscape of oncogenic signatures across human cancers. *Nature Genet,* 2013, 45: 1127-1130.

16. Cerami E, Gao J, Dogrusoz U, Gross BE, Sumer SO, Aksoy BA, et al. The cBio cancer genomics portal: an open platform for exploring multidimensional cancer genomics data. Cancer Discovery 2012, 2: 401-404.
17. Gao J, Aksoy BA, Dogrusoz U, Dresdner G, Gross B, Sumer SO, et al. Integrative analysis of complex cancer genomics and clinical profiles using the cBioPortal. Sci Signal. 2013, 6: pl1.

Supplementary Figure legends

Fig. S1. Expression ratio of cerebellar cell types between WT and *Jdp2* KO GCPs. (**A**) The GCPs were assigned based on the expression of *Atoh-1*, *Zic1*, *Pax6*, *NeuroD1*, and *Lhx9*. Purkinje cells were estimated based on the expression of *calbindin 1* and *2*, *Doc2b*, and *Pcp4*. Bergmann glial cells were estimated based on the expression of *Sept4* and *Cdf10*. Basket cells are represented by the expression of *Cck*, stellate cells by *Gria2*, astrocytes by *Cd44*, and oligodendrocytes by *Oligo2*. These expression frequencies were calculated as the total RNA expression and then summarized as a percentage of the relative RNA expression profiles in the cerebellum [1, 2]. The expression levels in oligodendrocytes, astrocytes, and Purkinje cells were higher in *Jdp2*-KO GCPs than they were in WT GCPs. In contrast, the expression of the markers in Golgi cells and GCs was lower in *Jdp2*-KO GCPs than it was in WT GCPs. (**B**) Western blot analysis of Cacna1a ($p21^{Cip1}$), Calbindin, Gabra1, Gabra6, Gabrb2, Grin2a (Glutamate [N-methyl-D-aspartate: NMDA] receptor subunit epsilon-1), PCP-4 (Purkinje cell protein-4) and Vglut1 (Vesicular glutamate transporter 1) proteins in Gabra6$^+$ PGCs cultivated with NAC for 7 days. The levels of expression of all markers were significantly higher in *Jdp2* KO vs. WT Gabra6$^+$ GCPs. The intensity of each band was then quantified. Uncropped raw data was shown in **Fig S5**. (**B**) Quantitative analysis of the results depicted in (A). The statistical analysis was performed as described in the Materials and Methods. The

expression of each protein in WT Gabra6^{+} GCPs was taken as 1.0 (n = 3; *P < 0.05; **P < 0.01; ***P < 0.001).

Fig. S2. Comparative expression of marker genes in WT and *Jdp2* KO Gabra6^{+}-PGCs that were cultured for 7 days with or without the addition of NAC. (**A**) Relative ARE-luciferase activities in WT and *Jdp2* KO Gabra6^{+}-GCPs cultured for 7 days in the presence or absence of NAC. ARE-luciferase activities were measured as described in the Materials and Methods. The ratio of ARE luciferase and pGL3 luciferase in WT PGCs cultured with NAC after the introduction of 50 and 100 ng of pGL3 luciferase was set as 1.0 (n = 3; ****P* < 0.001). (**B**) Expression of neural differentiation markers (GSK-3beta, NeuN, GABRA 6, and tubulin beta 3) in WT and *Jdp2* KO Gabra6^{+}-GCPs that were cultured for 7 days with NAC. **(C)** Immunostaining was performed as described in the Materials and Methods. These data were obtained from the data in Panel (B). The relative expression of tubulin beta 3, GSK3beta, NeuN, and Gabra6 in *Jdp2* KO GCPs was calculated in reference to that detected in WT Gabra6^{+}-GCPs, which was set as 1.0 (n = 5, **P* < 0.05; ***P* < 0.01; ****P* < 0.001).

Fig. S3. Summary of the data of the cBioPortal for Cancer Genomics. A total of 8,139 patients and 8,597 samples from 26 studies were summarized in the cBioPortal for Cancer Genomics (assessed at Sept. 21, 2022) [16-17]. (**A**) The sample and patients with overlapping counts of the GABRA-related genes in the altered and unaltered group are listed. The mutations of *GABRA1*, *GABRA6*, *GABRB2*, and *CD44* exhibited a greater number of overlapped counts. (**B**) The gene alteration frequency, the number of samples altered, and the percentages of alterations in the profiles of *GABRA1, GABRA6, GABRA2, GABRB2, CD44, SLC7A11, CDKN1A, NHE1L2*, and *JDP2* are listed. (**C**) Presentation of the co-occurrence tendency ratio of GABRA-related brain tumors. GABRA-related gene

expression was strongly correlated with *SLC7A11, CDKN1A,* and *NFE2L2*. Further details are provided in the Materials and Methods.

Fig. S4. Mutation profiles of GABRA1, GABRA6, GABRB2, CD44, SLC7A11, CDKN1A, NHE1L2, and JDP2. (**A**) Mutations, gene amplifications, and deep deletion profiles of *GABRA1, GABRA6, GABRB2, CD44, SLC7A11, CDKN1A, NHE1L2,* and *JDP2* genes. (**B**) Presentation of the mutation maps of members of the GABRA family, xCT-channel-related proteins, and proteins involved in the antioxidation response for the induction of neural differentiation. The cBioPortal data were accessed, and we surveyed the mutation maps of GABRA, xCT channels, GSH, and antioxidation-response-related proteins. In total, 8,139 patients and 8,597 samples from 26 studies were summarized in the cBioPortal for Cancer Genomics (assessed at Sept. 21, 2022) [15-17].

Fig. S5. Uncropped raw data of the Western blots that were used in this article. **(A–D)** Results are provided according to the sequence mentioned in the main text. **(A)** Fig. 1F **(B)** Fig. 2D, (**C**) Fig. S1B, (**D**) Fig. 6D. The red rectangles represent regions that were cropped for use in the figures of this text and in the supplementary data.

Supplementary Tables

STable 1; Antibodies used in this study

STable 2; Primers for qPCR used in this study

A

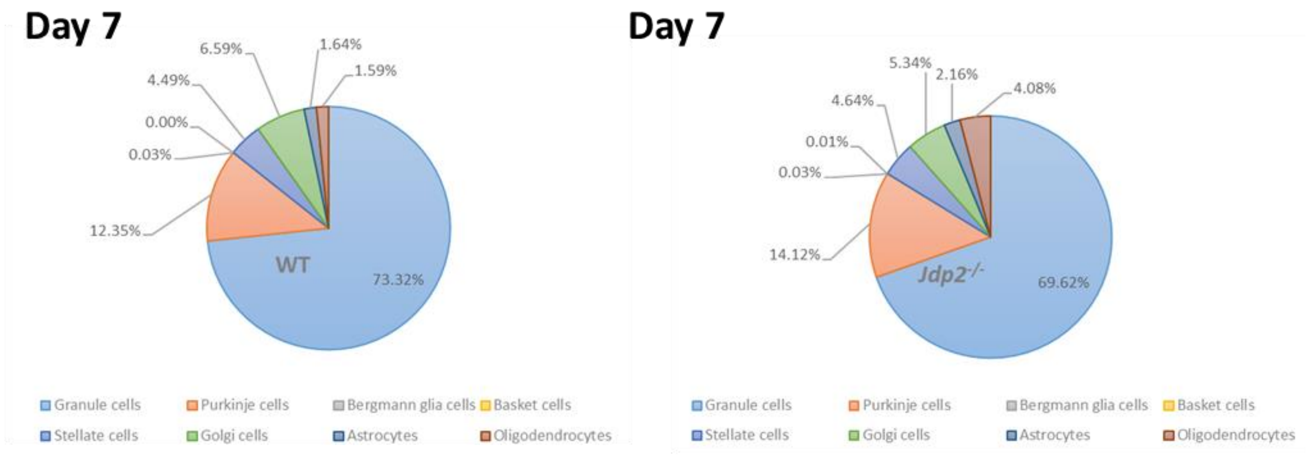
Day 7
6.59%
1.64%
1.59%
4.49%
0.00%
0.03%
12.35%
WT
73.32%
Granule cells
Purkinje cells
Bergmann glia cells
Basket cells
Stellate cells
Golgi cells
Astrocytes
Oligodendrocytes
Day 7
5.34%
2.16%
4.08%
4.64%
0.01%
0.03%
14.12%
Jdp2-/-
69.62%
Granule cells
Purkinje cells
Bergmann glia cells
Basket cells
Stellate cells
Golgi cells
Astrocytes
Oligodendrocytes

B

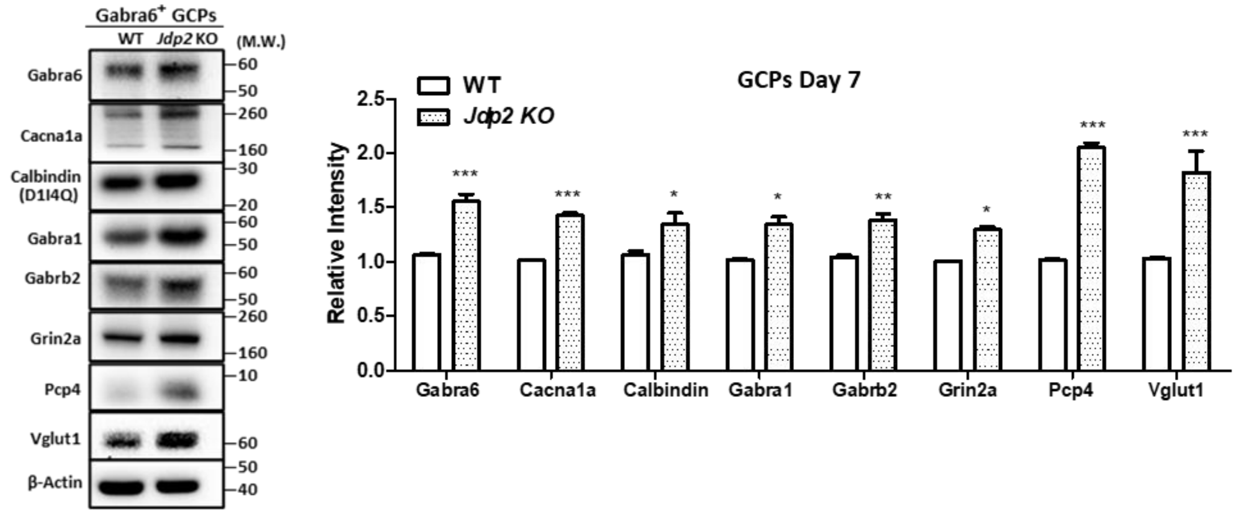
Gabra6+ GCPs
WT
Jdp2 KO
(M.W.)
Gabra6
Cacna1a
Calbindin (D1I4Q)
Gabra1
Gabrb2
Grin2a
Pcp4
Vglut1
β-Actin
60
50
260
160
30
20
60
50
60
50
260
160
10
60
50
40
WT
Jdp2 KO
GCPs Day 7
Relative Intensity
2.5
2.0
1.5
1.0
0.5
0.0
***
***
*
*
**
*
***
***
Gabra6
Cacna1a
Calbindin
Gabra1
Gabrb2
Grin2a
Pcp4
Vglut1

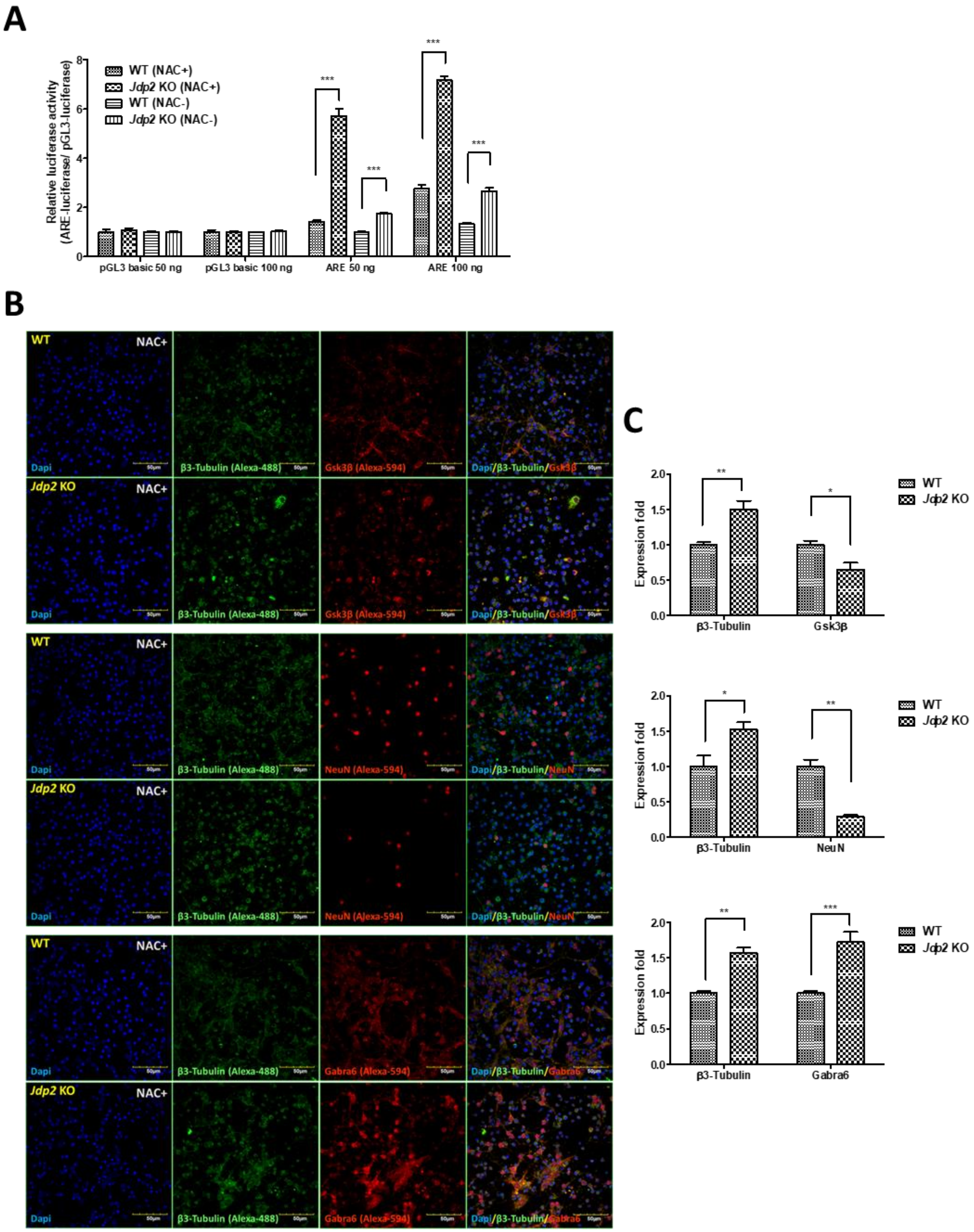
A
Relative luciferase activity
(ARE-luciferase/ pGL3-luciferase)
WT (NAC+)
Jdp2 KO (NAC+)
WT (NAC-)
Jdp2 KO (NAC-)
***
pGL3 basic 50 ng
pGL3 basic 100 ng
ARE 50 ng
ARE 100 ng
B
WT
NAC+
Jdp2 KO
Dapi
β3-Tubulin (Alexa-488)
Gsk3β (Alexa-594)
Dapi/β3-Tubulin/Gsk3β
NeuN (Alexa-594)
Dapi/β3-Tubulin/NeuN
Gabra6 (Alexa-594)
Dapi/β3-Tubulin/Gabra6
C
Expression fold
WT
Jdp2 KO
β3-Tubulin
Gsk3β
NeuN
Gabra6

A

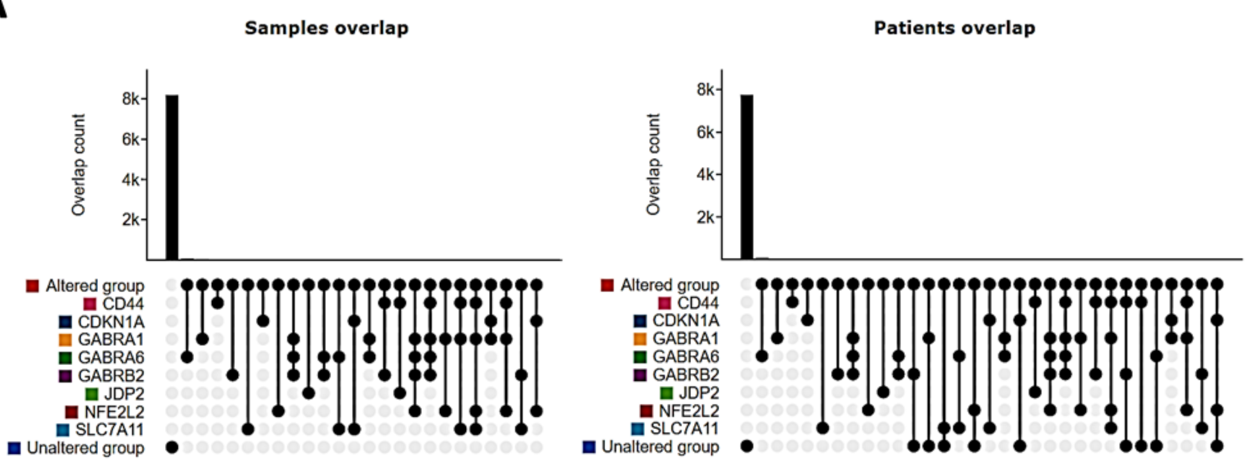

B

Gene Alteration Frequency

Gene Symbol	Num Samples Altered	Percent Samples Altered ▾
GABRA6	91	1%
GABRA1	74	<1%
GABRB2	69	<1%
CD44	42	<1%
SLC7A11	40	<1%
CDKN1A	34	<1%
NFE2L2	28	<1%
JDP2	13	<1%

Showing 1-8 of 8

C

A	B	Neither	A Not B	B Not A	Both	Log2 Odds Ratio	p-Value	q-Value ▴	Tendency
GABRA6	GABRB2	3918	53	36	22	>3	<0.001	**<0.001**	**Co-occurrence**
GABRA1	GABRA6	3907	47	59	16	>3	<0.001	**<0.001**	**Co-occurrence**
GABRA1	GABRB2	3922	49	44	14	>3	<0.001	**<0.001**	**Co-occurrence**
SLC7A11	CDKN1A	3976	32	18	3	>3	<0.001	**0.004**	**Co-occurrence**
GABRA1	NFE2L2	3957	60	9	3	>3	<0.001	**0.004**	**Co-occurrence**
GABRA1	CD44	3939	59	27	4	>3	0.001	**0.006**	**Co-occurrence**
CD44	NFE2L2	3988	29	10	2	>3	0.004	**0.014**	**Co-occurrence**
GABRB2	CD44	3943	55	28	3	2.941	0.010	**0.033**	**Co-occurrence**
GABRA6	SLC7A11	3922	72	32	3	2.352	0.026	0.082	Co-occurrence
SLC7A11	CD44	3965	33	29	2	>3	0.029	0.082	Co-occurrence
CD44	JDP2	3994	30	4	1	>3	0.038	0.096	Co-occurrence
SLC7A11	NFE2L2	3983	34	11	1	>3	0.100	0.222	Co-occurrence
GABRA1	SLC7A11	3933	61	33	2	1.966	0.103	0.222	Co-occurrence
NFE2L2	CDKN1A	4976	20	27	1	>3	0.111	0.222	Co-occurrence
GABRA1	CDKN1A	3946	62	20	1	1.670	0.282	0.527	Co-occurrence
GABRB2	SLC7A11	3937	57	34	1	1.023	0.399	0.699	Co-occurrence
GABRA6	CD44	3924	74	30	1	0.822	0.443	0.729	Co-occurrence

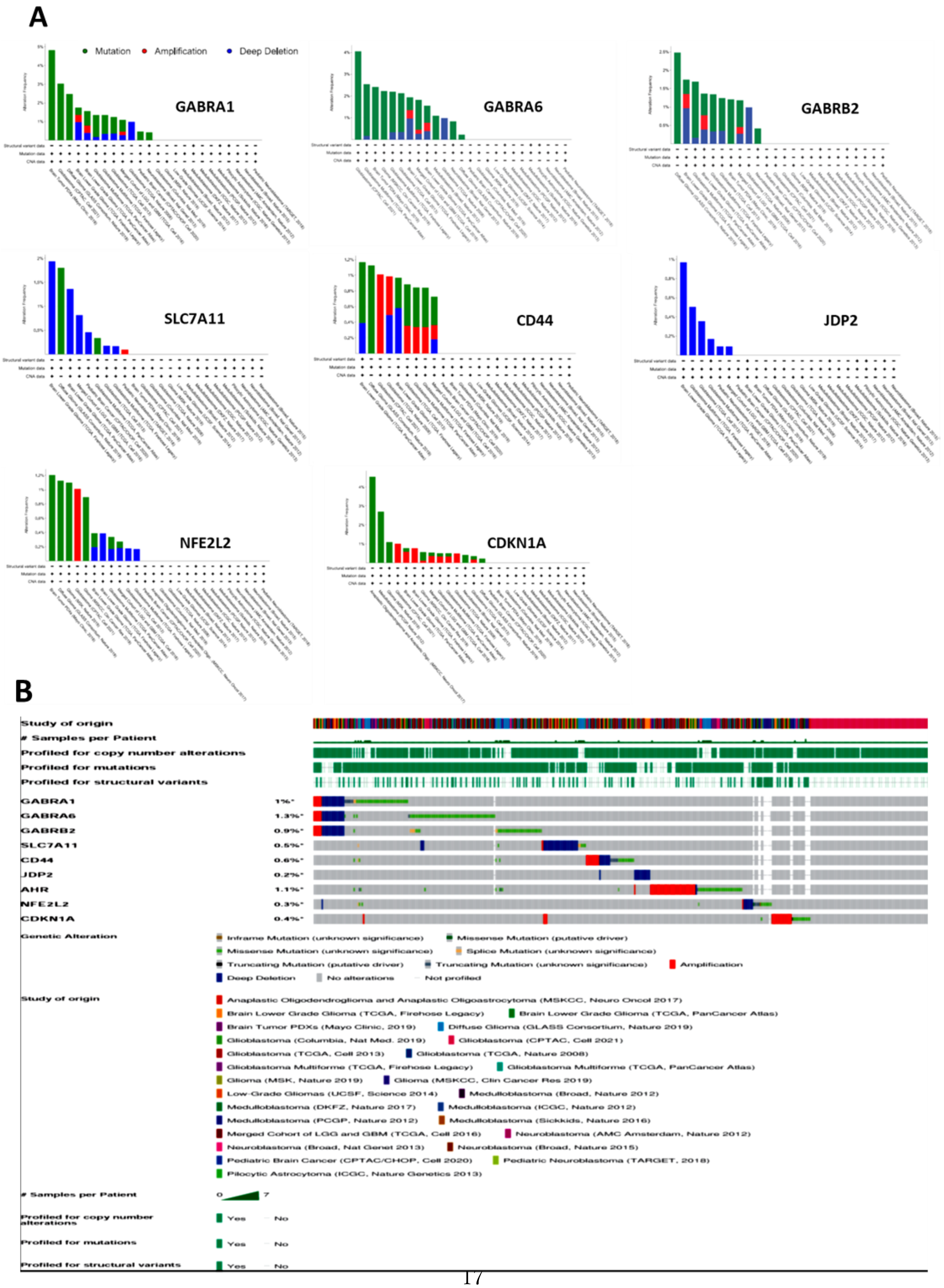
A
Mutation
Amplification
Deep Deletion
GABRA1
GABRA6
GABRB2
SLC7A11
CD44
JDP2
NFE2L2
CDKN1A
B
Study of origin
Samples per Patient
Profiled for copy number alterations
Profiled for mutations
Profiled for structural variants
GABRA1 1%*
GABRA6 1.3%*
GABRB2 0.9%*
SLC7A11 0.5%*
CD44 0.6%*
JDP2 0.2%*
AHR 1.1%*
NFE2L2 0.3%*
CDKN1A 0.4%*
Genetic Alteration
Inframe Mutation (unknown significance)
Missense Mutation (putative driver)
Missense Mutation (unknown significance)
Splice Mutation (unknown significance)
Truncating Mutation (putative driver)
Truncating Mutation (unknown significance)
Amplification
Deep Deletion
No alterations
Not profiled
Study of origin
Anaplastic Oligodendroglioma and Anaplastic Oligoastrocytoma (MSKCC, Neuro Oncol 2017)
Brain Lower Grade Glioma (TCGA, Firehose Legacy)
Brain Lower Grade Glioma (TCGA, PanCancer Atlas)
Brain Tumor PDXs (Mayo Clinic, 2019)
Diffuse Glioma (GLASS Consortium, Nature 2019)
Glioblastoma (Columbia, Nat Med. 2019)
Glioblastoma (CPTAC, Cell 2021)
Glioblastoma (TCGA, Cell 2013)
Glioblastoma (TCGA, Nature 2008)
Glioblastoma Multiforme (TCGA, Firehose Legacy)
Glioblastoma Multiforme (TCGA, PanCancer Atlas)
Glioma (MSK, Nature 2019)
Glioma (MSKCC, Clin Cancer Res 2019)
Low-Grade Gliomas (UCSF, Science 2014)
Medulloblastoma (Broad, Nature 2012)
Medulloblastoma (DKFZ, Nature 2017)
Medulloblastoma (ICGC, Nature 2012)
Medulloblastoma (PCGP, Nature 2012)
Medulloblastoma (Sickkids, Nature 2016)
Merged Cohort of LGG and GBM (TCGA, Cell 2016)
Neuroblastoma (AMC Amsterdam, Nature 2012)
Neuroblastoma (Broad, Nat Genet 2013)
Neuroblastoma (Broad, Nature 2015)
Pediatric Brain Cancer (CPTAC/CHOP, Cell 2020)
Pediatric Neuroblastoma (TARGET, 2018)
Pilocytic Astrocytoma (ICGC, Nature Genetics 2013)
Samples per Patient
0
7
Profiled for copy number alterations
Yes
No
Profiled for mutations
Yes
No
Profiled for structural variants
Yes
No

A Fig. 1F

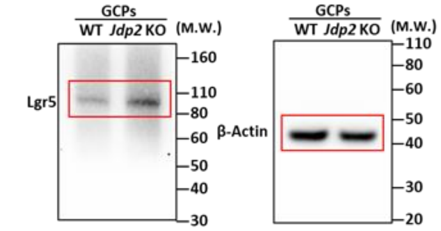

B Fig. 2D

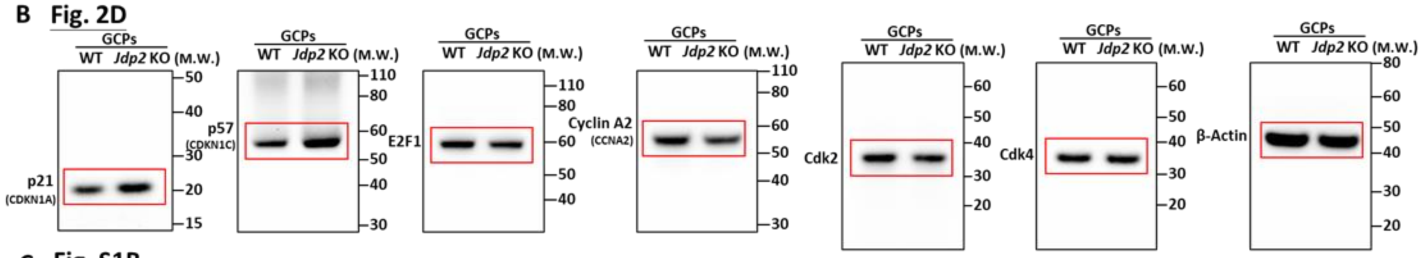

C Fig. S1B

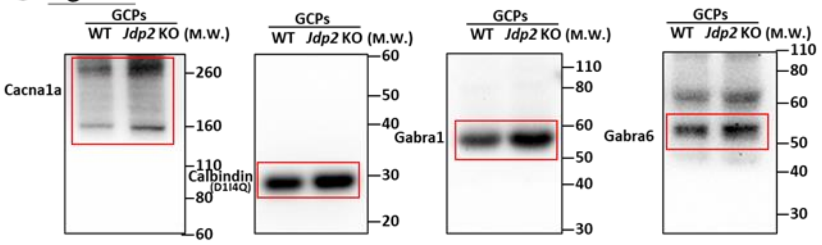

C Fig. S1B

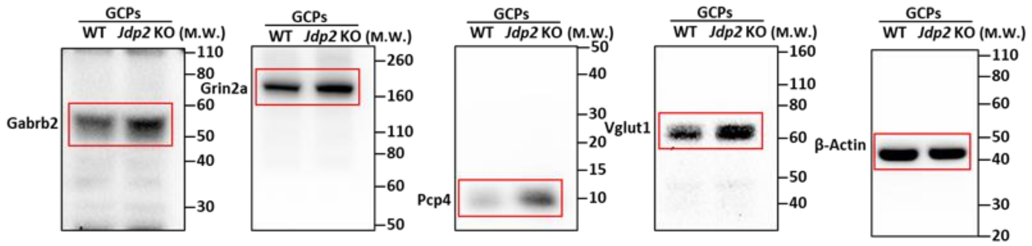

D Fig. 6D

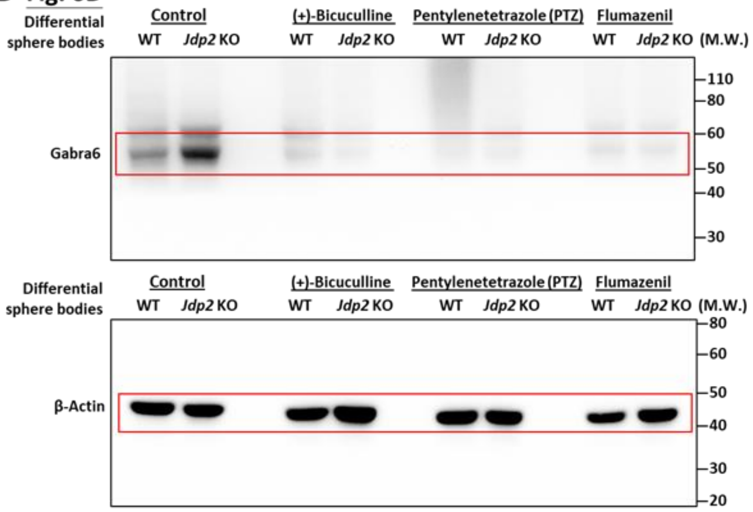

STable 1 Antibodies used in this study

Antibody name	Company	Cat. no.
Atoh1	Merck Millipore	AB5692
$p21^{Cip1}$	Santa Cruz Biotechnology	sc-397
AhR	Santa Cruz Biotechnology	sc-8088
Nrf2	Santa Cruz Biotechnology	sc-722
Nrf2	Cell Signaling Technology	CST #14596
p57	Santa Cruz Biotechnology	sc-56341
Cdk2	Santa Cruz Biotechnology	sc-70829
Cdk4	Santa Cruz Biotechnology	sc-260
E2F1	Santa Cruz Biotechnology	sc-193
CyclinA2	Santa Cruz Biotechnology	sc-596
Zic1	Merck Millipore	AB5786
Zic2	Merck Millipore	AB15392
Lgr5	Abcam	ab-75732
Lgr5	Bioss Inc	bs-1117R
Tubb3	Cell Signaling Technology	CST#4466
NeuN	Cell Signaling Technology	CST#24307
Calbindin	Cell Signaling Technology	CST #13176
Cacna1a	MyBioSource	mbs176970
Gabra1	R&D Systems	PPS022
Gabrb2	R&D Systems	PPS031
Gabra6	Bioss Inc.	bs-12063R
Gabra6	Bioss Inc	bs-12063R-FITC
CD45	Bioss Inc.	bs-10599R
Gsk-3β	Cell Signaling Technology	CST #9315
Neph3	R&D Systems	AF2930
Neph3	Novus Biologicals	NBP2-68977
Grin2a	R&D Systems	PPS012
Gclm	Biorbyt Ltd.	orb39793
Pcp4	MyBioSource	mbs2517367
Vglut1	Cell Signaling Technology	CST #12331
GFAP	Merck Millipore	04-1062
β-actin	Santa Cruz Biotechnology	sc-47778
Normal Rabbit IgG	Cell Signaling Technology	CST#2729

Anti-Rabbit-IgG HRP	Cell Signaling Technology	CST#7074
Anti-Mouse-IgG HRP	Cell Signaling Technology	CST#7076
Anti-Goat-IgG HRP	Santa Cruz Biotechnology	sc-2020
Anti-mIgGk HRP	Santa Cruz Biotechnology	sc-516102
Anti-Goat IgG HRP	Jackson ImmunoResearch Inc.	# 705-035-157
Anti-Rat-IgG HRP	Jackson ImmunoResearch Inc.	# 112-035-167
Alexa-Fluor® 488 conjugate Goat anti-Mouse IgG	Thermo Fisher Scientific	A-11029
Alexa Fluor® 488 conjugate Goat anti-rabbit IgG	Thermo Fisher Scientific	A-11034
Alexa Fluor® 594 conjugate Goat anti-mouse IgG	Thermo Fisher Scientific	A11032
Alexa-Fluor® 594 conjugate Goat anti-Rabbit IgG	Thermo Fisher Scientific	A-11037
Alexa Fluor® 647 Conjugate Goat anti-rat IgG	Cell Signaling Technology	CST#4418

STable 2 Primers for qPCR used in this study

qPCR primers	Primer sequences (5’-3’)	
mOct4	Sense	TCTTTCCACCAGGCCCCCGGCTC
	Antisense	TGCGGGCGGACATGGGGAGATCC
mKlf4	Sense	GCGAACTCACACAGGCGAGAAACC
	Antisense	TCGCTTCCTCTTCCTCCGACACA
mSox2	Sense	TAGAGCTAGACTCCGGGCGATGA
	Antisense	TTGCCTTAAACAAGACCACGAAA
mcMyc	Sense	TGACCTAACTCGAGGAGGAGCTGGAATC
	Antisense	AAGTTTGAGGCAGTTAAAATTATGGCTGAAGC
mNanog	Sense	TGGGTCTGCTACTGAGATGCTCTG
	Antisense	CAACCACTGGTTTTTCTGCCACCG
mGAPDH	Sense	ACCACAGTCCATGCCATCAC
	Antisense	TCCACCACCCTGTTGCTGTA
