# Supplementary figures and images for: Trans-differentiation of Jdp2-depleted Gaba-receptor-positive cerebellar granule cells to Purkinje cells
Source: Cell Death Discov. 2024 Dec 18;10:500. doi: 10.1038/s41420-024-02262-2 (PMC11655981; doi:10.1038/s41420-024-02262-2)

A Fig. 1F

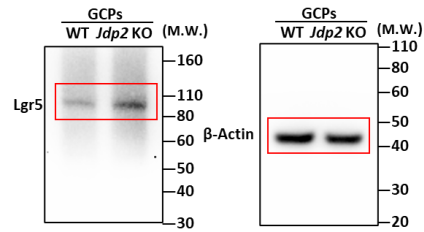

B Fig. 2D

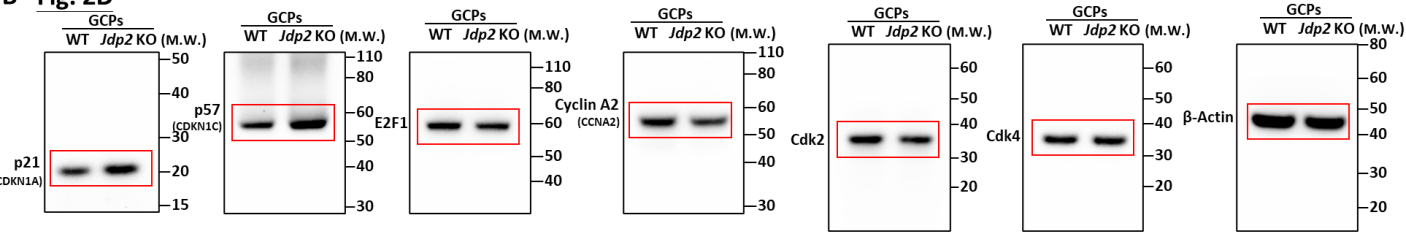

C Fig. S1B

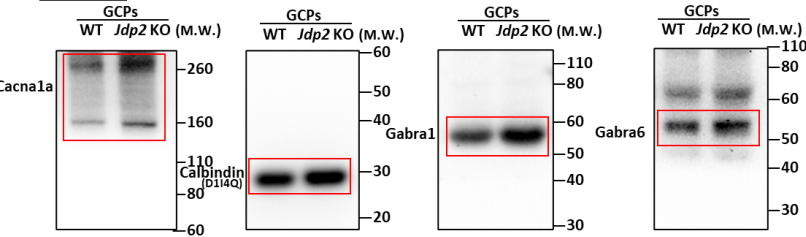

C Fig. S1B

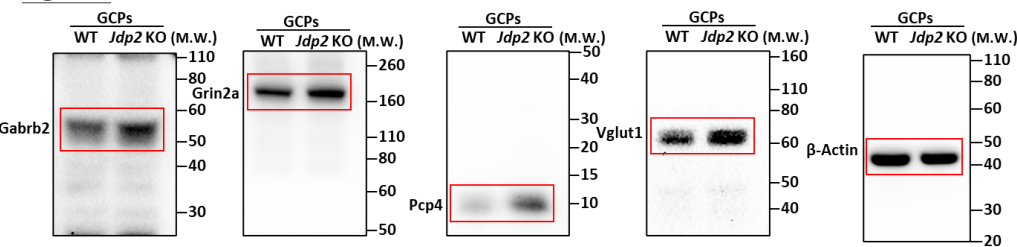

D Fig. 6D

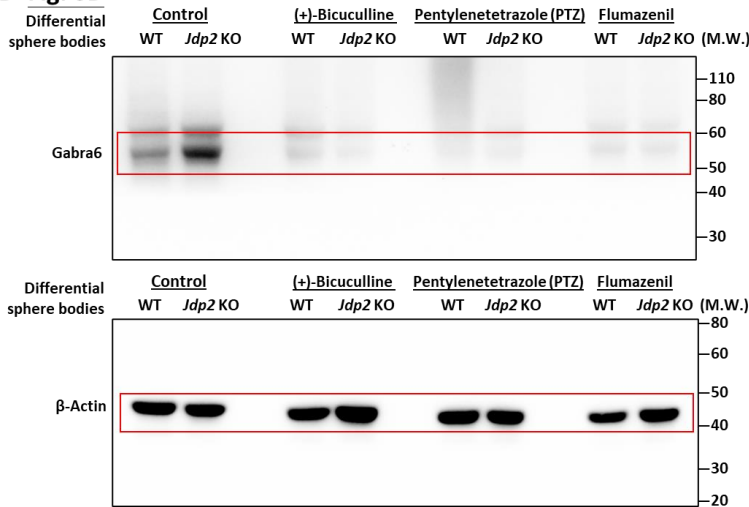

Supplement: Supplementary file 2 — Supplementary Figure 5, uncropped Western blots [file 41420_2024_2262_MOESM2_ESM.pdf]
